# The Yeast La Related Protein Slf1p Is a Key Activator of Translation during the Oxidative Stress Response

**DOI:** 10.1371/journal.pgen.1004903

**Published:** 2015-01-08

**Authors:** Christopher J. Kershaw, Joseph L. Costello, Lydia M. Castelli, David Talavera, William Rowe, Paul F. G. Sims, Mark P. Ashe, Simon J. Hubbard, Graham D. Pavitt, Chris M. Grant

**Affiliations:** 1 Faculty of Life Sciences, The University of Manchester, Manchester, United Kingdom; 2 Faculty of Life Sciences, Manchester Institute of Biotechnology (MIB), University of Manchester, Manchester, United Kingdom; MIT, United States of America

## Abstract

The mechanisms by which RNA-binding proteins control the translation of subsets of mRNAs are not yet clear. Slf1p and Sro9p are atypical-La motif containing proteins which are members of a superfamily of RNA-binding proteins conserved in eukaryotes. RIP-Seq analysis of these two yeast proteins identified overlapping and distinct sets of mRNA targets, including highly translated mRNAs such as those encoding ribosomal proteins. In paralell, transcriptome analysis of *slf1Δ* and *sro9Δ* mutant strains indicated altered gene expression in similar functional classes of mRNAs following loss of each factor. The loss of *SLF1* had a greater impact on the transcriptome, and in particular, revealed changes in genes involved in the oxidative stress response. *slf1Δ* cells are more sensitive to oxidants and RIP-Seq analysis of oxidatively stressed cells enriched Slf1p targets encoding antioxidants and other proteins required for oxidant tolerance. To quantify these effects at the protein level, we used label-free mass spectrometry to compare the proteomes of wild-type and *slf1Δ* strains following oxidative stress. This analysis identified several proteins which are normally induced in response to hydrogen peroxide, but where this increase is attenuated in the *slf1Δ* mutant. Importantly, a significant number of the mRNAs encoding these targets were also identified as Slf1p-mRNA targets. We show that Slf1p remains associated with the few translating ribosomes following hydrogen peroxide stress and that Slf1p co-immunoprecipitates ribosomes and members of the eIF4E/eIF4G/Pab1p ‘closed loop’ complex suggesting that Slf1p interacts with actively translated mRNAs following stress. Finally, mutational analysis of *SLF1* revealed a novel ribosome interacting domain in Slf1p, independent of its RNA binding La-motif. Together, our results indicate that Slf1p mediates a translational response to oxidative stress via mRNA-specific translational control.

## Author Summary

All organisms must respond to changes in their external environment such as exposure to different stresses. The availability of genome sequences and post-genomic technologies has enabled the analysis of these adaptive responses at the molecular level in terms of altered gene expression profiles. However, relatively few studies have focused on how cells regulate the translation of mRNA into protein in response to stress, despite its fundamental role in gene expression pathways. In this study, we show that a previously identified RNA-binding protein called Slf1p plays a major role in mRNA-specific regulation of translation during oxidative stress conditions and is necessary to promote the translation of stress-responsive mRNAs. This protein is a member of the so-called “La-related” family of proteins that have not been well characterized, although they are conserved throughout evolution. Exposure to oxidants is known to cause a general down-regulation of protein synthesis, although many stress response proteins are able to overcome this inhibition and increase their protein levels following stress by as yet unknown mechanisms. Our experiments offer one possible explanation, as they show that Slf1p plays a critical role in enhancing translation of many of these proteins, including many that are necessary for the cellular stress response.

## Introduction

The control of translation in response to external stimuli plays an important role in the regulation of gene expression. Indeed, some estimates of the relative contributions of different molecular mechanisms to the overall control of gene expression highlight a dominant role for translational control [1], [2]. Inhibition of translation initiation in particular forms a focus for much of this regulation. For example, in response to external stimuli, such as amino acid starvation or hydrogen peroxide stress, global translation initiation is normally reduced whilst significant numbers of specific mRNAs continue to be translated [3]. A variety of mechanisms exist to reduce the translation of most mRNAs e.g. through eIF2α phosphorylation, matched with complementary mechanisms to allow certain mRNAs to escape such global controls. One mechanism described to facilitate escape from global controls is via upstream ORFs; for example on the *GCN4* and *ATF4* mRNAs in yeast and mammals, respectively. In addition to intrinsic mRNA properties, a large number of RNA binding proteins (RBPs) are known to bind specific mRNAs in order to either activate or repress their translation [4], forming a cellular network of post-transcriptional regulation above that exerted at the transcriptional level.

Over 600 proteins encoded by the yeast genome are predicted to bind RNA [5] but the mechanisms by which RBPs control the translation of subsets of mRNAs are not yet clear. The La-motif (LaM) is an RNA binding domain which defines a superfamily of RNA-binding proteins conserved across eukaryotes [6]. Most organisms generally possess a true La protein ortholog with a LaM and one or more adjacent RNA-recognition (RRM) domains, which function in the nucleus binding RNA polymerase III primary transcripts. Human La was first identified as an autoantigen in patients suffering from autoimmune disorders. In addition there are a larger number of La related proteins (LARPs) most of which share the conserved adjacent LaM and RRM domains, but these proteins function in diverse processes, with both human LARP1 and LARP4 being implicated in binding polyA mRNAs and ribosomes [6]–[9].

*S. cerevisiae* has three LaM proteins; Lhp1p, Slf1p and Sro9p. Lhp1p is a true La protein ortholog, while Slf1p and Sro9p are atypical-LARPs that have a central LaM but lack any currently known RRM [10]. Slf1p and Sro9p appear evolutionarily most closely related to the LARP1 and 4 families [6]. In common with LARP1 and 4 family proteins, Slf1p and Sro9p preferentially associate with translating ribosomes and the polyA binding protein [7]–[11] and are believed to stimulate protein synthesis and/or promote mRNA stability of their bound mRNAs. Slf1p and Sro9p are homologous, sharing 30% identity at the amino acid level, but outside of the La domain there is little sequence similarity between Lhp1p and Slf1p/Sro9p. Cells deleted for *SRO9* display a slight slow growth phenotype, although null alleles lacking *SRO9, SLF1* and *LHP1* do not show any additive effects suggesting that they are not functionally redundant [10], [12]. Taken together, these data suggest that Slf1p and Sro9p are not required for protein synthesis but may have a role in the regulation of translation, possibly in an mRNA-specific manner. Interestingly, the *SLF1* mRNA, but not the *SRO9* mRNA, has a Puf3p binding site in its 3′-untranslated region, via which Puf3p is believed to repress translation of the *SLF1* mRNA [13]. As Puf3p primarily binds many mRNAs encoding mitochondrial functioning proteins this suggests that Slf1p may also have a role in mitochondrial function. Increased *SLF1* mRNA translation is also thought to promote respiration and the extension of yeast chronological life span.

To gain insight into the functions of this intriguing protein family, we have investigated the roles of the yeast LARPs using a full range of genome-scale techniques, including at the transcriptome, translatome and quantitative proteome level. Our studies have revealed a key role for Slf1p in the activation of translation of mRNAs critical for reprogramming gene expression to facilitate the cellular response to oxidative stress. We show that Slf1p has a critical role in mediating the coordinated cellular oxidative stress response to reactive oxygen species and *SLF1* is required for resistance to oxidative stress. In addition, mutational analysis of Slf1p reveals that it does have a novel ribosome-interaction domain independent of its mRNA binding LaM. Taken together our results provide a system-wide analysis of the role of the LARP Slf1p in an important cellular defence mechanism, highlighting it as a key player in the translational control of gene expression under oxidative stress conditions.

## Results

### Transcriptome analysis of *sro9Δ* and *slf1Δ* strains suggests that Sro9p has less impact on steady state mRNA levels than Slf1p

**Figure 1:**
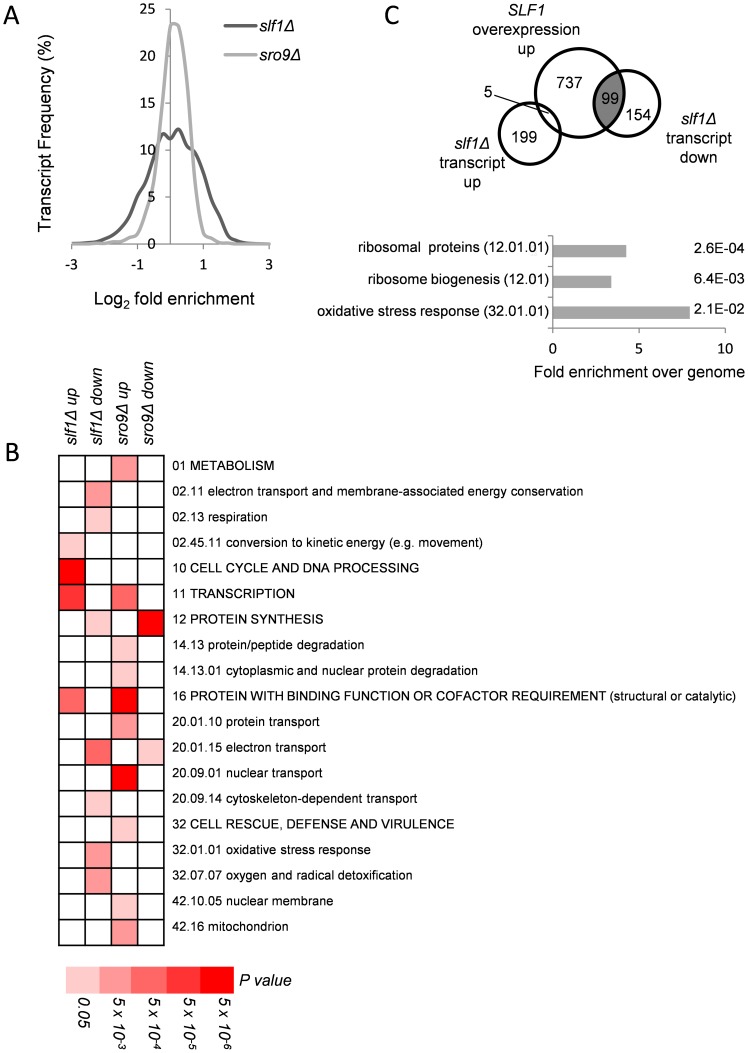
Alterations in mRNA abundance in *slf1* and *sro9* deletion mutants. (**A**) Relative transcript abundance changes are shown for the *slf1Δ* and *sro9Δ* mutant strains, determined by RNA-Seq, compared to the parental strain and expressed as Log_2_ fold enrichment. Transcriptome changes were split into bins (0.25 fold/bin) and expressed as a percentage of transcripts in each bin. (**B**) Functional categorisation of those transcripts whose abundance is altered in *slf1Δ* or *sro9Δ* mutant strains. Results are ordered on MIPS category classification numbers and overarching categories are in capitals. Where an overarching category was enriched, sub-categories within the overarching category were omitted from the graph. Confidence of each classification category is shown as Bonferroni corrected p-values. (**C**) Venn diagram comparing transcripts that alter after deletion of *SLF1* with the transcriptome of a strain overexpressing *SLF1* [12] and MIPS categorisation of the shaded crossover is shown, again confidence is shown as Bonferroni corrected p-values.

Slf1p and Sro9p are related LARPs that both associate with translating ribosomes. We therefore decided to assess their roles in RNA biology using a range of post-genomic techniques. Slf1p and Sro9p share 37% overall amino acid identity, 57% within the LaM, suggesting they likely have similar or overlapping roles. We used RNA sequencing (RNA-Seq) to assess the impact of deletion of each LARP independently, by comparing the relative total transcript levels in *slf1Δ* and *sro9Δ* mutant strains with an isogenic wild-type strain. Triplicate samples were processed using a standard workflow (see Materials and Methods) that revealed 204 mRNAs were significantly increased (FDR<0.05) and 253 mRNAs decreased in abundance compared to the parental strain in an *slf1Δ* mutant (S1 Table and S1A Fig.). In comparison, an *SRO9* deletion mutant showed a greater impact, with 702 mRNAs increased and 666 mRNAs decreased in an *sro9Δ* strain (S2 Table and S1A Fig.). Although more transcripts alter following loss of *SRO9*, in general the degree of change appears more modest with only 35 transcripts increasing and 85 decreasing by more than 2 fold. In contrast the variation in fold change is much greater in the *slf1Δ* strain (Fig. 1A).

RNA-binding proteins that mediate post-transcriptional control can interact with functionally related mRNAs [14]. We therefore searched for functional categories enriched among the differentially expressed *slf1Δ* and *sro9Δ* mRNAs using MIPS category classifications. Classes including respiration, protein synthesis and the oxidative stress response were statistically over represented among *slf1Δ* down-regulated transcripts, while only protein synthesis was similarly affected following *sro9Δ* (Fig. 1B). There are 141 mRNAs down-regulated by both gene deletions suggesting some overlap in the targets of each LARP and as expected they are enriched in genes involved in protein synthesis according to MIPS (Fig. 1B). Thus, both Sro9p and Sfl1p appear to contribute to the regulation of mRNAs involved in protein synthesis, while Slf1p has targets in additional pathways. The functional classes enriched in transcripts that were up-regulated following the loss of each factor are largely distinct (Fig. 1B), as expected, since fewer mRNAs (only 71) were up-regulated in both datasets (S1B Fig.) suggesting that these proteins influence the mRNA architecture of the cell in a related but distinct manner.

A recent study investigated the effect of overexpressing *SLF1* on mRNA abundance and identified 852 mRNAs that increase in abundance and 599 mRNAs that decrease in abundance using a microarray-based approach [12]. We compared this dataset with our *slf1Δ* dataset and found a highly significant overlap between those mRNAs that increase in abundance when *SLF1* is overexpressed and those mRNAs that decrease in abundance in an *slf1Δ* strain (Fig. 1C). Indeed, the 99 transcripts that appear in both experiments are significantly enriched for ribosome biogenesis, ribosomal proteins and oxidative stress response functions (Fig. 1C).

The functional categories of transcripts that alter in abundance in an *sro9Δ* strain are different compared with those in the *slf1Δ*, with the exception of transcription (up-regulated) and protein synthesis (down-regulated). When comparing the two transcriptomes to each other, there are 71 transcripts that increase in both the *slf1Δ* strain and the *sro9Δ* strain and 141 transcripts that decrease in abundance in both strains (S1B Fig.). However, there is also a modest crossover between the transcript sets that increase in one of the two mutant strains but decrease or don't change in the other (S1B Fig.). Beyond the shared role of the yeast LARPs in regulating mRNAs involved in protein synthesis, particularly mRNAs encoding ribosomal proteins, we noted Slf1p's additional potential role in mediating the responses to oxidative stress. Since it has been shown previously that Slf1p promotes copper detoxification [12], which is related to oxidative stress tolerance and which require regulations and reprogramming of protein synthesis [15], it is this role that we explore further in this study.

### RIP-Seq identification of Slf1p and Sro9p target mRNAs

We developed a rapid RIP-Seq approach to identify RNAs bound by TAP-tagged proteins, using strains bearing genomically-integrated C-terminal TAP tags. Our strategy involved minimally disturbing cells and processing them as rapidly as possible to maintain physiological interactions. This used swift cell freezing in liquid nitrogen and cell lysis, followed by an immunoprecipitation step using IgG conjugated to paramagnetic beads. Using paramagnetic beads enabled rapid immunoprecipitation and washes and resulted in sample processing that generated significantly reduced background binding in comparison to approaches relying on extended incubations such as cross-linking protocols or employing agarose beads which are prone to non-specific interactions. In our protocol total RNA and RNA isolated from the Slf1p-TAP and Sro9p-TAP immunoprecipitated fractions was depleted for rRNA and then converted into cDNA sequencing libraries using standard methods (see Materials and Methods).

**Figure 2:**
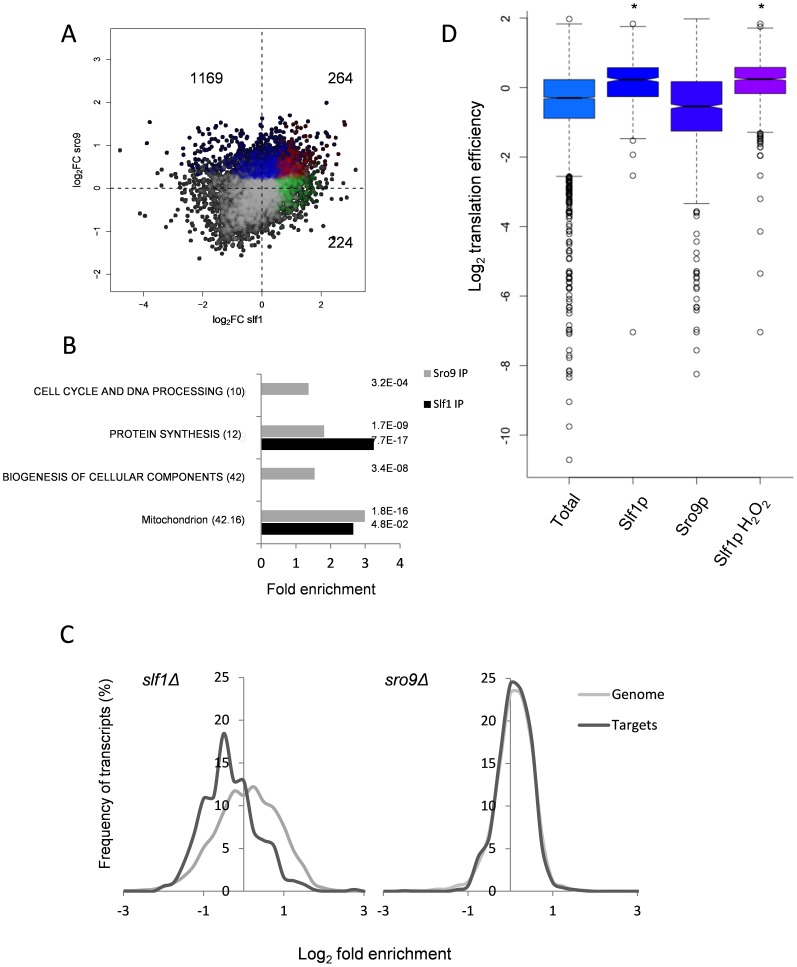
Comparison of Slf1p and Sro9p target mRNAs. (**A**) A scatterplot comparing the mRNA targets identified in the Slf1p (green), Sro9p (blue) or in both (red) Rip-Seq experiments. The number of ORFs identified as unique to Slf1p or Sro9p or in both are indicated. (**B**) MIPS Functional categorisation of Slf1p and Sro9p target mRNA enrichment. (**C**) Slf1p maintains steady state levels of its mRNA targets. Transcript abundance of the whole transcriptome and target mRNAs of Slf1p (Left) or Sro9p (Right) were analysed as described in the legend to Fig. 1A. Slf1p and Sro9p targets were filtered (FDR<0.05). The x axis of the graph has been restricted to show only those data that are in bins between 3 and -3. (**D**) Translation efficiency [16] of mRNAs bound in each IP and the total RNA. Outliers are shown (open circles) and samples with a P<2.2 -e16 (Wilcoxon rank) are indicated (asterisk).

Triplicate Slf1p-TAP and Sro9p-TAP RIP-Seq experiments identified 488 and 1433 mRNAs, respectively, that are significantly enriched above total RNA (corrected FDR<0.05) (S3–S4 Tables). When the two datasets were compared, only 264 transcripts were identified as being significantly enriched by both Slf1p and Sro9p (Fig. 2A). Despite this, a Gene Ontology analysis of the independent Slf1p and Sro9p mRNA-target sets shows common enrichment above the genomic background for mRNAs involved in protein synthesis and mitochondrial functions (Fig. 2B). Functional analysis of the 264 transcripts bound by both Slf1p and Sro9p also identified enrichment for protein synthesis and mitochondrial functions. Sro9p mRNA targets were also enriched for ‘cell cycle and DNA processing’ and ‘biogenesis of cellular components' categories. During the course of our study, mRNA targets for both Slf1p and Sro9p were additionally reported by an independent study using a RIP-Chip approach [12]. Although the overlap between the studies appears to be modest, a significant number of transcripts are common to both datasets and the functional classes enriched are the same (S2 Fig.). Both studies identify protein synthesis, particularly mRNAs encoding ribosomal proteins as significant targets of Slf1p and Sro9p.

When comparing functionally enriched gene classes of the Slf1p and Sro9p mRNA targets (Fig. 2B) with those mRNAs that change transcriptionally in the corresponding mutant strains (Fig. 1B), the enrichment in common functional themes is further reinforced. Genes linked to protein synthesis are both transcriptionally down-regulated in the deletion strains and bound by both factors. Similarly, genes within the ‘mitochondrion’ MIPS category are enriched in both Sro9p targets and are up-regulated in the *sro9Δ* mutant. We therefore examined the specific overlap in transcripts between the two sets, comparing transcriptionally regulated genes with targets identified in our RIP-Seq experiment. Notably, Slf1p-mRNA targets are also down-regulated in the *slf1Δ* mutant (56 mRNAs, *P* = 3.67×10^−11^; Fisher's Exact test), whereas there is little crossover with those mRNAs (6 mRNAs) that increase in abundance (S3A Fig.), suggesting that Slf1p is required for maintaining steady state target mRNA levels. In contrast, there were far fewer than expected Sro9p-TAP bound mRNAs whose transcript levels are altered in an *sro9Δ* mutant (S3B Fig.). The origin of this effect is clearly evident in Fig. 2C, which shows the distribution of log2 transcriptional fold changes in the deletion strains, highlighting the distributions of transcripts also bound by the equivalent TAP-tagged protein in the RIP experiment; Slf1p targets are clearly less abundant in *slf1Δ* cells while Sro9p target abundance is apparently unchanged. Applying increasing FDR cut off stringencies to our Rip-Seq data to restrict our analysis to the most significant hits maintains these trends (S4 Fig.).

To gain further insight into Slf1p and Sro9p functions, we compared the RIP-Seq targets with other recently published genome wide measurements of mRNA half-life, PolyA tail length and ribosome occupancy by ribosome footprinting [16]. The only significant finding was that Slf1p mRNA targets are enriched for mRNAs that are actively translated and therefore have a higher translational efficiency (Fig. 2D). We conclude that the LARPs bind both overlapping and distinct sets of mRNAs including highly translated mRNAs such as those encoding ribosomal proteins. Unexpectedly, loss of Sro9p does not significantly alter mRNA target levels, while loss of Slf1p does. This suggests that Slf1p targets may be under greater dynamic control than those bound by Sro9p, or that other factors can more easily compensate for loss of Sro9p than for Slf1p.

### *SLF1* is required for growth under oxidative stress conditions

**Figure 3:**
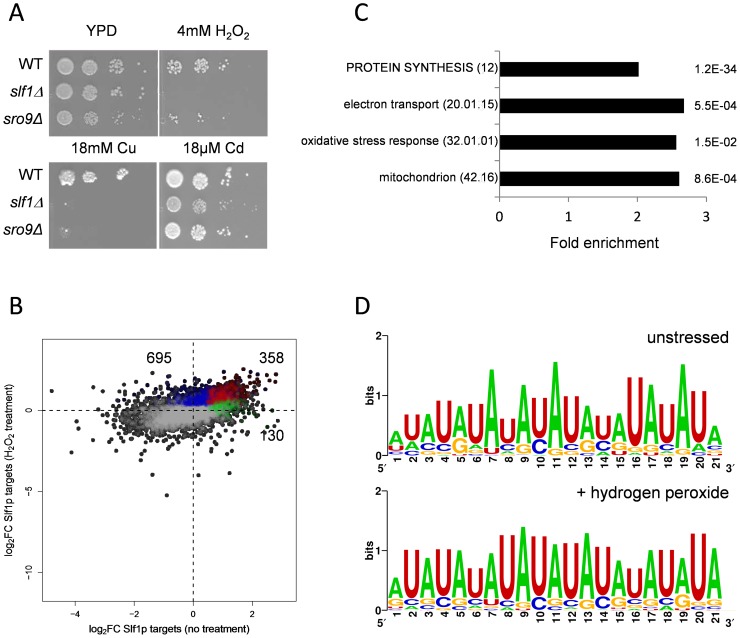
Slf1p and Sro9p are required for growth under oxidative stress conditions. (**A**) Growth of the wild-type, *slf1Δ* and *sro9Δ* mutant strains on the indicated media for 3 days at 30°C. (**B**) Scatterplot comparing the mRNA targets identified by Slf1p-RIP Seq under control conditions (green) compared with 15 minutes of treatment with 0.4 mM Hydrogen peroxide (blue). ORFs identified under both conditions are indicated (red). The numbers of ORFs present in each group are shown (**C**) Functional categorisation of those mRNAs that are enriched in the Slf1p RIP Seq after peroxide treatment. Category classification is presented, as described in the legend to Fig. 1. (D) 3′ UTR motifs of mRNAs bound by Slf1p in the presence and absence of hydrogen peroxide identified using the MEME Suite [43].

Our transcriptome analyses suggest a role for *SLF1* in mediating the oxidative stress response. We further examined this finding by testing the sensitivity of *slf1Δ* and *sro9Δ* mutants to a range of stress conditions. We first confirmed that the growth of both mutant strains is inhibited by copper as previously described [17] and found that *slf1Δ* mutants, and to a lesser extent *sro9Δ* mutants, are sensitive to hydrogen peroxide stress (Fig. 3A). Mutants deleted for *SLF1* also showed a modest sensitivity to cadmium, which like hydrogen peroxide causes oxidative stress (Fig. 3A). Sensitivity to stress conditions is not a general property of *slf1Δ* and *sro9Δ* mutants since little or no sensitivity was found with various other stress conditions, including growth at elevated or lower temperatures (37°C or 16°C), at pH 5, or high salt (1 M NaCl) (S5A Fig.). Plasmid-borne *SLF1* complements the *slf1Δ* mutant sensitivity to hydrogen peroxide, confirming that Slf1p is important for oxidative stress tolerance (S5B Fig.).

### Slf1p immunoprecipitates mRNAs required for oxidative stress tolerance following treatment with hydrogen peroxide

Our data indicate that Slf1p is important for oxidative stress tolerance. Because many stress responsive genes are transcriptionally and/or translationally activated in response to stresses such as hydrogen peroxide [15], [18] we assessed Slf1p RNA targets by RIP-Seq following treatment of cells with 0.4 mM hydrogen peroxide, a concentration sufficient to induce a robust and rapid reprogramming of gene expression [15]. Hydrogen peroxide treatment increased the number of significantly bound mRNAs to 1053 compared to 488 in the untreated Slf1p RIP-Seq experiment (S5 Table). Reassuringly, there was still a highly significant overlap between both datasets and 358 transcripts were bound by Slf1p during normal and oxidative stress conditions (Fig. 3B). Functional enrichment analysis of the stress-bound mRNAs again highlighted the oxidative stress response, mitochondrial function, electron transport and protein synthesis as significantly enriched MIPS categories (Fig. 3C). The expanded set of RNA-targets retain very high ribosome occupancy (Fig. 2D). This suggests that Slf1p is binding actively translated mRNAs that are required for the cellular response to oxidative stress.

### Slf1p is associated with actively translating ribosomes during oxidative stress conditions

**Figure 4:**
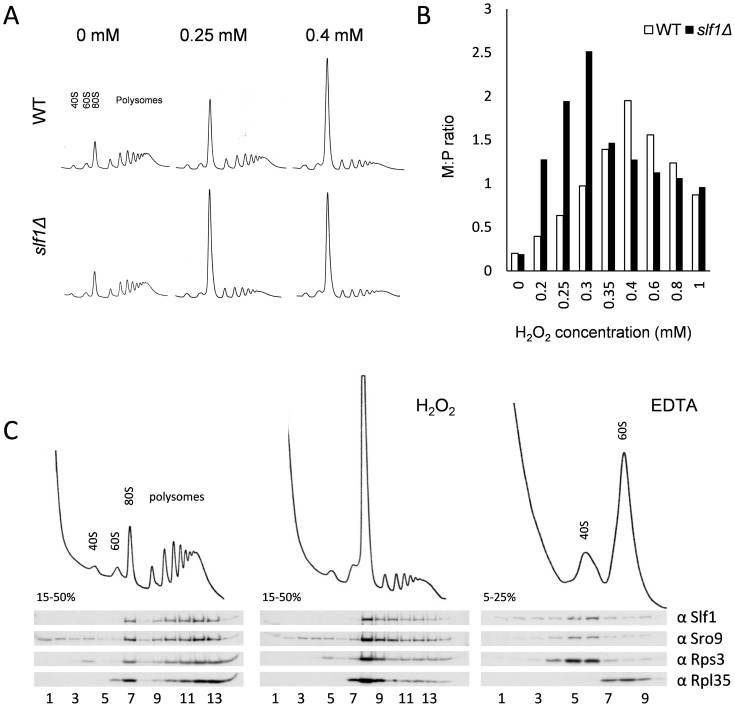
Slf1p is associated with actively translating ribosomes during oxidative stress conditions. (**A**) Polyribosomal profiles of the *slf1*Δ and wild-type strains before or after hydrogen peroxide treatments for 15 min. (**B**) Quantification of the ratio of ribosomes in monosomes (80S) to Polysomes (M:P) over a 0–1 mM range of hydrogen peroxide concentrations. The polyribosomal profiles which were used to generate this data are shown in S6 Fig. (**C**) Ribosome-association of both Slf1p-TAP and Sro9p in fractions isolated from sucrose gradients of an Slf1p-TAP tagged strain. Cultures were treated with 0.4 mM hydrogen peroxide for 15 minutes or with EDTA as shown.

Both Sro9p and Slf1p associate with translating ribosomes [10] and oxidative stress is known to cause a global reprogramming of protein synthesis [15], [19]. Our data show that under these conditions, Slf1p binds oxidative stress regulated mRNAs and that *slf1Δ* cells are sensitive to oxidative stress conditions. We therefore investigated the impact of *SLF1* and *SRO9* deletions on the global translational response to hydrogen peroxide stress using polyribosomal profiling. Deletion of *SLF1* does not affect the polyribosome profile in unstressed cells (Fig. 4A). In contrast, following a 15 minute treatment with 0.25 mM hydrogen peroxide, the *slf1Δ* strain exhibited a more dramatic inhibition of translation initiation than the parental strain; as detected by an increase in the 80S monosome peak compared to the polysome ribosomal peaks (Fig. 4A). A similar but less pronounced effect was observed for the *sro9Δ* strain (S5C Fig.). Repeating polyribosomal profiling experiments over a range of hydrogen peroxide concentrations (S6 Fig.) revealed that the *slf1Δ* mutant strain exhibits maximum translation inhibition at a lower hydrogen peroxide concentration than the wild-type strain (quantification shown in Fig. 4B). Based on these findings, we suggest that the enhanced inhibition of translation in response to oxidative stress conditions may account for the growth sensitivity of this mutant.

As noted previously, Slf1p and Sro9p co-sediment with ribosomal subunits across polysome gradients, suggesting that these proteins interact with actively translating ribosomes [10]. To track Slf1p and Sro9p across gradients, we used the Slf1-TAP strain employed in our RIP-Seq experiments. We confirmed that the addition of a TAP-tag does not affect the growth or stress sensitivity of the yeast strains (S5A Fig.). Treating cells with 0.4 mM hydrogen peroxide causes an inhibition of translation initiation, but Slf1p and Sro9p still associate with polysomes (Fig. 4C), indicating that they remain associated with the fraction of ribosomes still actively translating mRNAs.

### Slf1p and Sro9p co-immunoprecipitate members of the closed loop complex following stress

**Figure 5:**
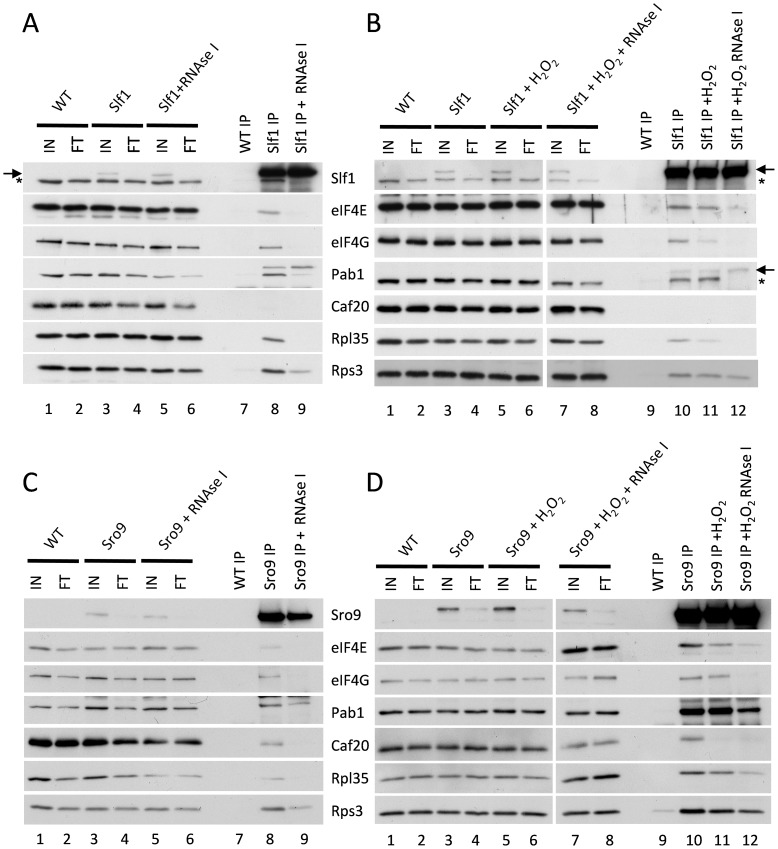
Slf1p and Sro9p immunoprecipitate members of the closed loop complex in an RNA-dependent manner. Slf1p-TAP (**A**) and Sro9p-TAP (**C**) co-immunoprecipitate eIF4E, eIF4G, Pab1p, Rps3p and Rpl35p. The immunoprecipitation of eIF4E, eIF4G and Rpl35p is RNA dependent as treatment with RNAse I during the immunoprecipitation prevents the co-immunoprecipitation of these proteins by both Slf1p-TAP (A) and Sro9p-TAP (C). This RNAse I treatment reduces, but does not eliminate, the co-immunoprecipitation of Pab1p and Rps3p by both Slf1p and Sro9p. Sro9p-TAP, but not Slf1p-TAP, co-immunoprecipitates the eIF4E binding protein Caf20p in an RNA dependent manner. Co-immunoprecipitations are also shown for Slf1p-TAP (**B**) and Sro9p-TAP (**D**) following treatment with 0.4 mM hydrogen peroxide for 15 minutes. Oxidative stress specifically affects the Slf1p-TAP-eIF4G (B) and Sro9p-TAP Caf20p (D) interactions. Bands corresponding to Slf1-TAP (arrow) and Pab1p (asterisk) in panels A and B arise due to re-probing the same blots for Slf1p and Pab1p.

Cap-dependent eukaryotic translation initiation requires the eukaryotic translation initiation factors eIF4E and eIF4G and is enhanced by the poly(A) binding protein, Pab1p. The cap is bound by eIF4E and Pab1p binds the poly(A) tail of the mRNA. The scaffold protein eIF4G binds to both eIF4E and Pab1p forming a ‘closed loop’ complex that is thought to promote protein synthesis [20]. If our hypothesis that the yeast LARPs remain associated with actively translating mRNAs following stress is correct, these translation factors should also be associated with the LARPs following stress. To test this, we used the Slf1p-TAP and Sro9p-TAP strains and performed TAP affinity purifications and Western blotting with specific antibodies to assess whether translation initiation factors remain associated with each LARP. In purifications from unstressed cells, both LARPs immunoprecipitated a fraction of key closed-loop proteins eIF4E, eIF4G and Pab1p, as well as markers for the 40S (Rps3p) and 60S (Rpl35p) ribosomal subunits (Fig. 5A lane 8 and 5C lane 8). This is consistent with previous work identifying interactions between Slf1p and eIF4E or Pab1p [21]. However, RNase I treatment diminished co-immunoprecipitation of the closed loop factors with both LARPs (Fig. 5 panels A and C, lanes 9) implying that these interactions are mRNA mediated.

Repeating the experiments following treatment with 0.4 mM hydrogen peroxide for 15 minutes, largely maintained these interactions (Fig. 5 B and D, comparing lanes 10 and 11), although the interaction of eIF4G with Slf1 appears more sensitive to hydrogen peroxide than the other factors. Again these interactions were RNase I sensitive (Fig. 5 B and D compare lanes 11 and 12). In summary, the interactions of both LARPs with initiation factors that are components of the closed loop complex, as well as ribosomal proteins, suggests that they interact with actively translated mRNAs following stress.

### Slf1p activates translation of oxidative stress response proteins

**Figure 6:**
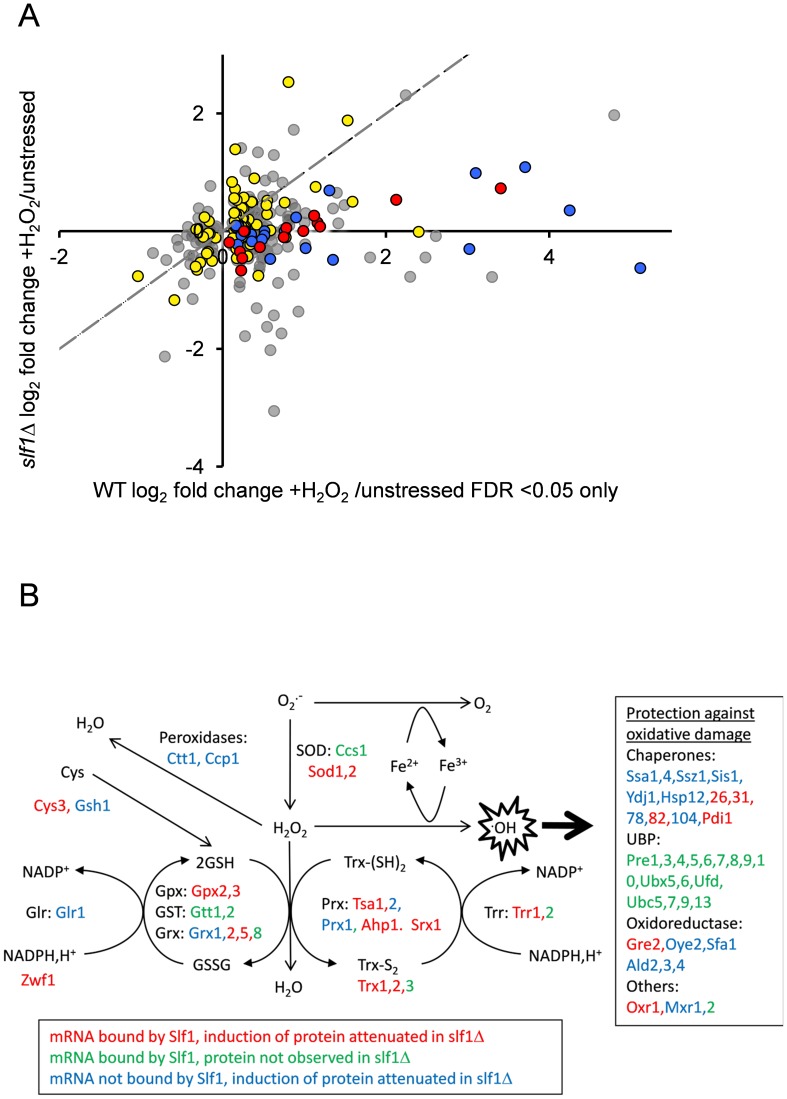
Slf1p is required for oxidative stress gene expression during oxidative stress conditions. (**A**) The fold-enrichment change is shown for those proteins identified as increasing or decreasing in the wild-type after peroxide treatment compared with the *slf1Δ* mutant. All proteins on the scatter plot were found to significantly alter in abundance (FDR<0.05) in the wild-type strain following oxidative stress (315 proteins; 249 up and 66 down). Proteins encoded by Slf1p target mRNAs are indicated as red and yellow dots. These include proteins which form part of the oxidative stress response according to MIPS (red dots) as well as proteins which are not directly involved in the oxidative stress response (yellow dots, for details see text). Proteins which form part of the oxidative stress response but are not direct Slf1p targets are shown as blue dots. The dotted line shows the trend-line that would be expected if there was no difference between the wild-type and *slf1Δ* mutant. (**B**) Diagrammatic representation of the oxidative stress response highlighting changes in the *slf1Δ* strain. Hydrogen peroxide (H_2_O_2_) is generated by the breakdown of superoxide (O_2_^.-^) catalysed by superoxide dismutases (SOD). Hydrogen peroxide can be reduced by iron (Fe2+) in the Fenton reaction to produce the highly reactive hydroxyl radical (^.^OH). Various antioxidant enzymes are involved in the defence against hydrogen peroxide including peroxidases, peroxiredoxins (Prx), glutathione peroxidases (Gpx), glutathione transferases (GST), glutaredoxins (Grx), thioredoxins (Trx), glutathione reductase (Glr), thioredoxin reductase (Trr) and glutathione (GSH). mRNAs bound by Slf1p where protein induction is attenuated in the *slf1Δ* are in red (corresponding to red dots in Fig. 6A), mRNAs bound by Slf1p where the corresponding protein was not detected in the *slf1Δ* are in green and mRNAs which are not bound by Slf1p, but where protein induction is attenuated in the *slf1Δ* are in blue (corresponding to blue dots in Fig. 6A).

Our data so far, strongly suggest that Slf1p has a significant role in promoting or protecting the translation of genes necessary for the cellular response to oxidative stress. If so, we reasoned that at least some of the oxidative stress induced changes in gene expression manifest at the translational level would be dependent upon Slf1p. Therefore, to examine oxidative stress induced proteome changes, we used a label-free quantitative mass spectrometry (LC-MS) approach comparing the total cell extract proteome during normal growth conditions and following addition of hydrogen peroxide. Five replicate wild-type stressed and unstressed samples were analysed, enabling quantitation of 1565 proteins in the wild type strain (see Materials and Methods and S6 Table), of which 315 altered significantly (249 up and 66 down) in response to peroxide stress (FDR p<0.05). Significantly, 97 of these are encoded by Slf1p mRNA targets identified by our RIP-Seq following oxidative stress. By repeating the proteome analysis in an *slf1Δ* strain, we identified 2140 proteins, of which only 2 increased in abundance significantly (FDR p<0.05) in response to hydrogen peroxide (S7 Table), suggesting that the oxidative stress induced reprogramming of the proteome is significantly muted in *slf1Δ* cells. It is possible that that some of the decrease in bulk translational activity in the *slf1* mutant might arise due to decreased mRNA abundance in the mutant strain. However, of the 248 proteins which showed an attenuated protein induction in the *slf1* mutant, only 33 were found to decrease at the mRNA level in the *slf1* mutant strain. Of the 97 proteins identified as altered by oxidative stress in the wild-type strain that are encoded by Slf1p mRNA targets, 83 of these proteins were also quantified in our *slf1Δ* proteomics experiment (red and yellow symbols in Fig. 6A). Fourteen of these proteins are involved in the oxidative stress response (red symbols in Fig. 6A) as are a further 22 proteins that are not encoded by Slf1p-mRNA targets (blue symbols in Fig. 6A). It is clear from the plot in Fig. 6A that the induction of oxidative stress related proteins is significantly attenuated in *slf1Δ* cells (red and blue symbol positions deviate significantly below the dotted X = Y line shown in Fig. 6A).

Comparing Slf1p mRNA-targets identified following stress conditions with the proteome data obtained with wild-type cells identified 109 proteins induced by oxidative stress that are encoded by Slf1p mRNA targets. Although 100 of these proteins were also identified and quantified in our *slf1Δ* proteomics experiment, only 13 significantly increased in abundance after peroxide stress in a *slf1Δ* strain. Importantly, functional classification of the 87 proteins that were no longer stress induced in the mutant strain showed significant enrichment for proteins involved in the oxidative stress response and detoxification and repair of oxidant damage (red dots, Fig 6A), highlighting Slf1p's role in mediating translation of these key transcripts.

Fig. 6B shows an overview of the antioxidants and stress repair and detoxification proteins which comprise the yeast oxidative stress response. Proteins indicated in red correspond to Slf1p-mRNA targets where their oxidative stress protein induction is attenuated in an *slf1Δ* strain (red circles in Fig. 6A). These include a number of key antioxidants including superoxide dismutase (Sod1p and Sod2p), thioredoxins (Trx1p and Trx2p), thioredoxin reductase (Trr1p), peroxiredoxins (Tsa1p, Ahp1), glutaredoxin (Grx2p, Grx5p), glutathione peroxidases (Gpx2p, Gpx3p) and the stress protective enzyme sulfiredoxin (Srx1p). A number of proteins were also identified where their oxidative stress induction was attenuated in the *slf1Δ* mutant but they were not identified as direct mRNA targets of Slf1p in the RIP-Seq analysis. These proteins are indicated in blue on Fig. 6B (and blue circles in Fig. 6A). Additional proteins are highlighted which were identified as Slf1p-mRNA targets but where we were unable to detect any high confidence peptide identifications for their parent proteins in the proteomics analysis (Fig. 6B, in green). When taken together our series of ‘omics studies reveal the importance of Slf1p in mediating translational control of the expression of key oxidative stress genes. We propose that Slf1p activates translation of its target mRNAs.

### Slf1p interacts with ribosomes through a domain independent of the LaM

To gain more insight into the mechanism of Slf1p regulation of protein synthesis, we further examined its ribosome binding activity. Treating whole cell extracts with EDTA dissociates 80S monosomes and polyribosomes into 40S and 60S ribosomal subunits, and we noted that Slf1p-TAP and Sro9p both co-sediment with a small ribosomal subunit marker, Rps3p (Fig. 4C). Similarly, in our TAP-IP experiments, interactions between each LARP and Rps3p appear resistant to RNase I treatment (Fig. 5, lanes + RNase). These studies suggest that both yeast LARPs interact with 40S ribosomal subunits in an RNA independent manner.

**Figure 7:**
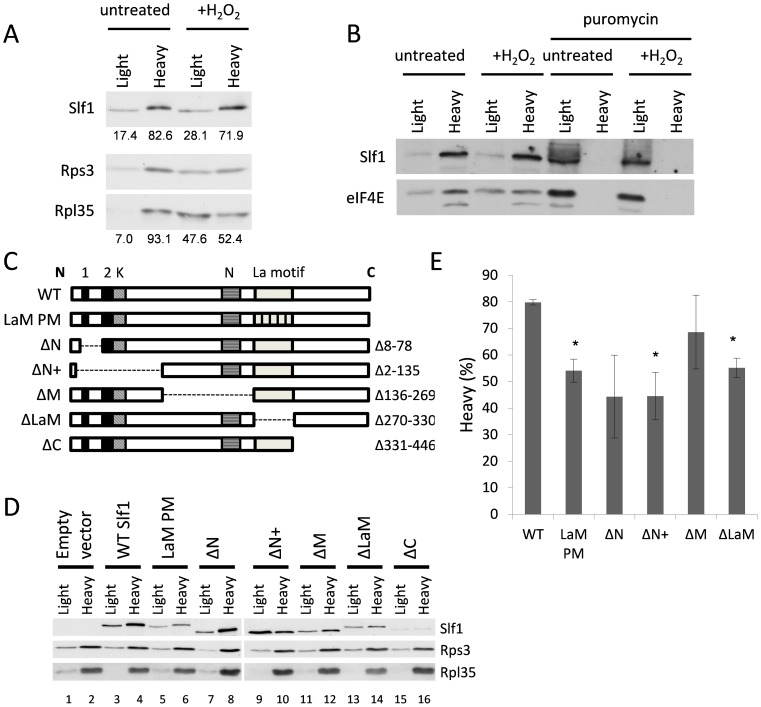
Identification of regions in Slf1p required for its ribosome-interaction. (**A**) Sucrose cushion gradient fractions separated by SDS PAGE and Slf1p, Rpl35p and Rps3p detected by immunoblot analysis. (**B**) Sucrose cushion gradients are show as for panel (A) except extracts were treated with 1 mg/ml puromycin prior to loading onto gradients. Slf1p and eIF4E were detected by immunoblot analysis. (**C**) Diagrammatic representation of Slf1p and the constructs made. The LaM (270–330) and conserved regions A (residues 30–42), B (residues 77–92) (Black boxes), K (lysine rich region from 92–124) and N (asparagine rich region between 172–234) (hatched boxes). The La-PM allele combines F281A, Y282A, F293A and F314A mutations. (**D**) Sucrose cushion analysis of Slf1p deletion mutants, as in panel A. (**E**) Quantification of sucrose cushion gradients. Error is shown as standard error of the mean for three biological repeats. Data where p<0.02 are indicated (*).

To further examine Slf1p-ribosome association, we used a sucrose cushion assay, which is simpler than a full polysome analysis and useful for screening purposes. Here, cell lysates were resolved into light and heavy fractions on sucrose cushion gradients. In untreated cells, Slf1p is mainly present in the heavy ribosome associated fraction along with the majority of the 40S and 60S ribosomal subunit markers (Fig. 7A). After treatment with hydrogen peroxide (0.4 mM, 15 min), although a significant proportion of the 40S and 60S ribosomal subunit markers shifted from the heavy ribosome-associated fraction into the lighter fraction, Slf1p is retained in the heavy fraction (Fig. 7A). Coupled with Fig. 4, we interpret these results as suggesting that, in this assay, ribosomes associated with mRNA remain in the heavy fraction while mRNA-free 80S monosomes are present in the lighter fraction. As a further proof that Slpf1p associates with actively translating ribosomes, cell extracts were treated with puromycin prior to separation using sucrose cushion assays [22], [23]. Puromycin is an aminonucleoside antibiotic that causes premature chain termination during translation and the collapse of translating heavy polysomes. Puromycin caused a shift in the distribution of Slf1p from the heavy to light fractions in cell extracts from both control and peroxide treated cells (Fig. 7B). Similarly, the initiation factor eIF4E was shifted from heavy to light fractions in response to puromycin treatment. This is consistent with Slf1p associating with actively translating ribosomes in both control and stressed yeast cells.

A previous study identified the Slf1p LaM as necessary for mRNA binding [12]. Outside the LaM, Slf1p has no other recognised domains. Sequence alignments reveal two short regions towards the N-terminus with similarity to Sro9p and their homologs in other yeasts, along with two separate lysine and asparagine rich sequences (Fig. 7C). To determine if the ribosome association of Slf1p similarly relies upon the LaM or if other regions are important, five truncation mutants of Slf1p were constructed deleting different regions of *SLF1* (Fig. 7C). In addition, a missense allele was created where four LaM residues key for RNA binding [24] are altered to alanines in the context of the full length protein (LaM-PM). Each construct was C-terminally TAP tagged and introduced into *slf1Δ* cells as the sole source of Slf1p. The sucrose cushion assay was used to analyse the impact of each mutation on the interaction of Slf1p with the ribosome (Fig. 7D and E). Deletion of the Asn-rich region between the amino terminus and the La motif (ΔM) had little effect on ribosome association. Deletion of the extreme C-terminus (ΔC) of Slf1p was difficult to interpret since it significantly reduced the levels of Slf1p and so was not considered further. In contrast, both N-terminal deletions (ΔN, ΔN+) and La motif mutations (ΔLaM, and LaM PM) significantly decreased the level of Slf1p present in the heavy fraction compared with the corresponding wild type (Fig. 7D and E). This experiment suggests that both of these regions are functionally important in maintaining Slf1p with mRNA-associated ribosomes.

**Figure 8:**
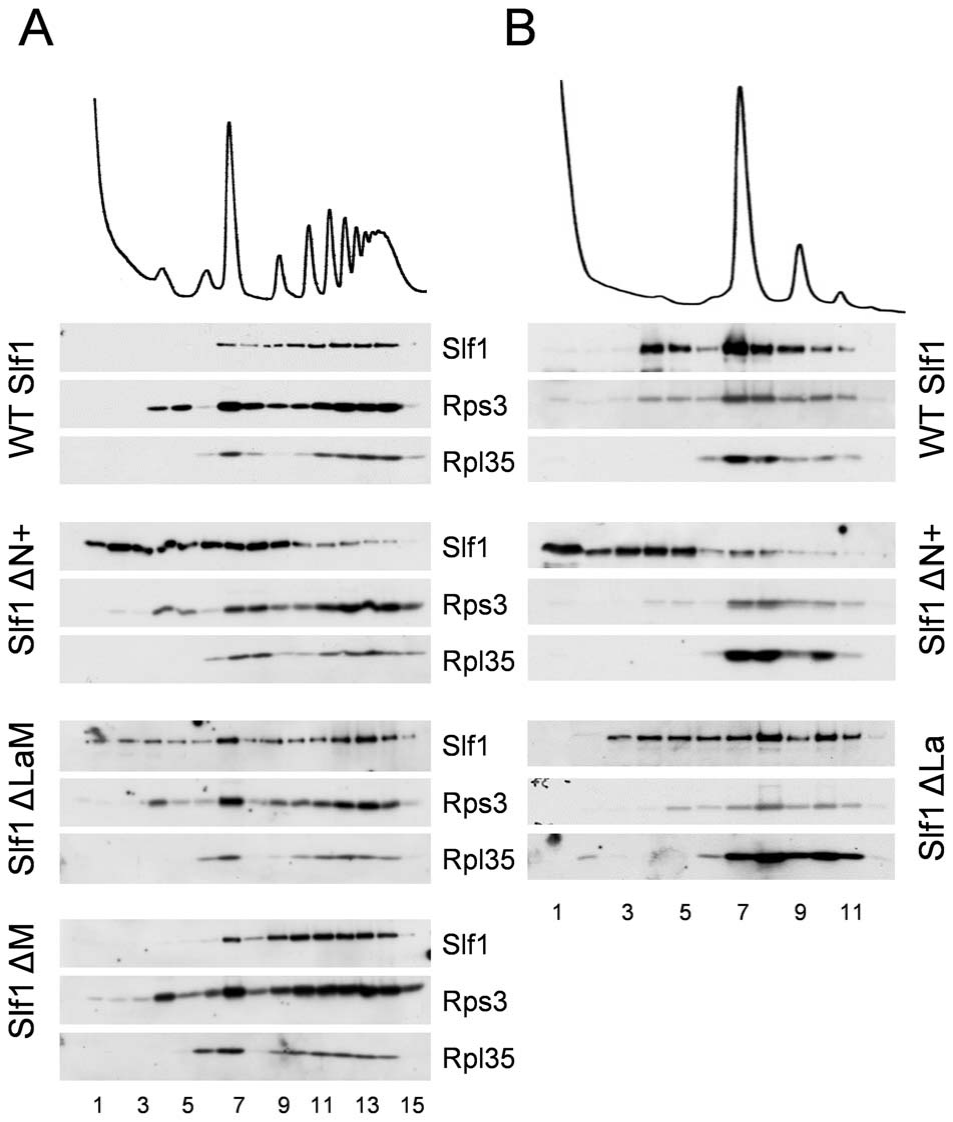
Slf1p associates with ribosomes independently of the La motif. (**A**) Western blotting of fractions isolated from polyribosome gradients are shown for strains expressing *SLF1*, ΔN+, ΔLaM and ΔM mutants. (**B**) As (A) but with RNAse I treated extracts.

As a more robust test of our interpretations, the sedimentation of both ΔN+ and ΔLaM constructs were assessed across full polysome gradients. Their sedimentation patterns were significantly altered relative to that of the wild type protein, with a higher proportion co-sedimenting in light fractions away from the ribosomal material (Fig. 8A). As a control, the ΔM deletion was observed to co-sediment with the translating ribosomes similar to wild type Slf1p (Fig. 8A). These data indicate that there may be two ways that Slf1p can interact with the ribosome; through its N-terminal region or through the LaM. The LaM likely acts to promote the interaction of Slf1p with ribosomes indirectly via its interaction with mRNA [12]. We tested this idea by RNAse I treatment to disrupt polysomes and re-assessed the ribosomal-association of the Slf1p mutants (Fig. 8B). Following RNase I treatment, wild type Slf1p remained associated with the resulting 80S ribosomes and ΔLaM had a modest impact on Slf1p ribosome association. In contrast, however, removal of the N terminal region (ΔN+) significantly disrupted ribosome binding by Slf1p. Taken together with other data presented here these findings are consistent with idea that there are separable functional domains within Slf1p: the N terminus of Slf1p acting as a 40S ribosome binding domain, whereas the La motif facilitates mRNA interaction.

## Discussion

We set out to characterize the roles of the yeast LARPs Slf1p and Sro9p via an integrated set of post-genomic global analyses. These experiments have confirmed that both these homologous RNA-binding proteins have similar functional roles, with overlapping sets of mRNA targets that they bind and regulate in terms of abundance at the mRNA level, including many ribosomal proteins (Fig. 1 and 2). Slf1p target mRNAs are among the most actively translated mRNAs, identified by ribosome profiling (Fig. 2D). This and other data strongly suggests Slf1p is a translational activator.

Our experiments reveal that Slf1p has a critical role in mediating the coordinated cellular oxidative stress response to reactive oxygen species. Several lines of evidence show that Slf1p remains bound to actively translating mRNAs during oxidative stress (Fig. 4, 5 and 7) and that some of the stress mRNA targets encode many key antioxidant enzymes including thioredoxins, glutaredoxins and peroxiredoxins that are all critical to the cellular defence against hydrogen peroxide and whose expression is enhanced following stress (Fig. 3 and 6). Oxidative stress leads to a general down-regulation of protein synthesis initiation, caused by phosphorylation of eIF2, as well as defects in the elongation phase of protein synthesis [15]. Yet, stress response proteins are apparently able to overcome this inhibition and increase or maintain their protein levels following stress by as yet unknown mechanisms. Our experiments offer one possible explanation, as they show that Slf1p plays a critical role in enhancing translation of many of these proteins, including many that are necessary for the cellular stress response (Fig. 6). As a consequence, *slf1Δ* cells are hyper-sensitive to hydrogen peroxide both in terms of growth and overall protein synthesis, as measured by polysome profiles (Fig. 3 and 4).

Finally, we present evidence that Slf1p binding to the small ribosomal subunit is not solely dependent on the LaM, but instead optimally requires a novel motif within the N-terminal region of Slf1p (Fig. 7 and 8). Thus, we suggest that Slf1p acts as an adapter protein between specific mRNAs and the ribosome, promoting translation of key mRNAs during stress conditions by binding both 40S ribosomes (via the N-terminal ribosome binding domain) and specific target mRNAs (via the LaM), with both domains critical for resistance to ROS.

Notably, Sro9p is approximately six times as abundant as Slf1p according to Pax-DB [25], which is reflected both by the increased number of its target mRNAs and the increased number of mRNAs whose levels are altered in its absence, although the change in abundance observed is generally less than two-fold. Intriguingly, and despite this, Slf1p has a greater impact on steady state mRNA levels of its targets than does Sro9p. Slf1p-target mRNAs are reduced in abundance in *slf1Δ* cells, while Sro9p does not significantly influence its target mRNA abundance. This provides further support to the idea that these LARPs are not functionally equivalent despite sharing many mRNA targets. Specificity may be achieved by binding other distinct partners. Distinctions between the yeast LARPs that we identified were that Sro9p (i) forms an RNA-dependent complex with Caf20p, while Slf1p does not, and, (ii) that Pab1p interaction with Sro9p appears less sensitive to RNase than does the Pab1p-Slf1p interaction In addition hydrogen peroxide treatment apparently reduced levels of eIF4G binding Slf1p. Possible implications for these observations are described below.

Efficient translation of mRNAs involves capping of the 5′-end and polyadenylation of the 3′-end of the mRNA. The 5′ methyl cap is bound by eIF4E, the polyA tail is bound by multiple Pab1p proteins and eIF4G binds to both eIF4E and Pab1p forming a ‘closed-loop’ complex that is thought to promote translation [20]. As expected for factors promoting translation and interacting with ribosomes, Slf1p and Sro9p co-immunoprecipitate initiation factors which are part of the closed-loop complex, as well as components of the small and large ribosomal subunits. These interactions are largely RNA dependent. Caf20p competes for the eIF4G binding site of eIF4E, preventing the formation of the closed-loop complex and thus suppressing translation of certain mRNAs [26],[27]. RNA-dependent co-purification of Caf20p with Sro9p may indicate that some of the mRNAs bound by Sro9p are not translationally active. In accord with this idea, a proportion of the Sro9p signal was found migrating in non-ribosomal fractions of polysome gradients. The Sro9p-Caf20p interaction is reduced following hydrogen peroxide stress although we do not know if this is significant. Similarly Sro9p-Pab1p interactions appear more resistant to RNase than Slf1p-Pab1p interactions. This may imply direct binding between Sro9p and Pab1p. We have not explored this possibility further, although studies of related proteins found similar interactions. The human LARPs 4 and 4b were also found to bind 40S subunits and PABP (polyA binding protein) [8], [11]. LARP4 interacts with PABP through a PABP interaction motif 2 (PAM2) found in its extreme N-terminus and which is shared with some other unrelated PABP interacting proteins and a second region downstream of the LaM and RRM domains [8]. It remains to be determined whether the continued mRNA-dependent interaction between the cap-binding complex factors and Slf1p under oxidative stress conditions, reflects Slf1p remaining bound to actively translating mRNAs. An alternative possibility is that both the cap-binding complex and Slf1p remain bound to repressed mRNAs during oxidative stress conditions since translation initiation can be blocked at several distinct steps, some of which lie downstream of the cap-binding complex. The interaction between Slf1p and eIF4G is diminished following oxidative stress. At present the significance of this observation also remains unresolved. Interactions between Slf1p and Pab1p/eIF4E are maintained, suggesting that at least some mRNAs bound by Slf1p may specifically lose eIF4G after hydrogen peroxide stress. Slf1p does not directly bind to eIF4E after hydrogen peroxide treatment, as this interaction remains RNA dependent, ruling out the possibility that Slf1p acts as a direct eIF4E binding protein.

Our sucrose density gradient and immunoprecipitation data both clearly indicate that Slf1p and Sro9p associate largely with the 40S small ribosomal subunit in a manner that is resistant to EDTA and/or RNase treatment. In agreement with a previous study, the LaM [12] of Slf1p is responsible for mRNA binding, while here we identify a novel 40S binding domain in the Slf1p N-terminus that is shared with Sro9p and their close homolog's. This split in generic and specific recognition is not unprecedented in LARPs. The human LARPs 4 and 4b also have 40S ribosome interacting domains that are distinct to their LaM. The C-terminus of LARP4b was shown to interact with the 40S protein RACK1 [11]. LARP4 also binds to RACK1 [8]. RACK1 is located on the head of the 40S ribosomal subunit close to the mRNA exit channel [28] [29]. Therefore it is ideally placed to act as an adapter for RNA-binding proteins. In yeast RACK1 is called Asc1p and it is known to act as a ribosome binding site for the RBP Scp160p [30] and can regulate translation [31].

A recent study used genome-wide ribosome profiling to analyse the translational response to oxidative stress induced by hydrogen peroxide exposure [32]. This study provides translation efficiency (TE) data (amount of footprint normalized to underlying mRNA abundance) following treatments with 0.2 mM hydrogen peroxide for five or 30 minutes. We have compared this data with our RIP-Seq and proteomics analyses, which treated yeast cells with 0.4 mM hydrogen peroxide for 15 minutes. In order to investigate any possible association between the ribosome footprinting results and our RIP-Seq and proteomics analyses, following [32], we classified mRNAs and proteins as being up, down or unchanged in our experiments. S7 Fig. shows that the distribution of TE values is not the same across both the transcript and protein abundance subsets (Kruskal-Wallis test; FDR<0.01). In particular, we found an enrichment for mRNAs that are significantly down in the RIP-Seq experiment and have low TE following 30-minute stress (χ^2^ test; Bonferroni corrected p-value = 0.0009). In addition, there is also an association between proteins with increased abundance in the proteomics experiment and mRNAs with increased TE in the ribosome footprinting experiment after 5-minute stress (χ^2^ test; Bonferroni corrected p-value = 0.044), and 30-minute stress (χ^2^ test; Bonferroni corrected p-value = 0.034). In summary, the main conclusions from comparing these datasets are: 1) that being an Slf1p mRNA target does not increases TE under stress conditions, but protects against a decrease in TE; 2) that this effect does not appear to be immediate; and, 3) that mRNAs that have an increased TE under stress conditions, tend to have increased protein production.

It was recently shown that human LARP1 is necessary to enhance translation of 5′ TOP mRNAs [33]. 5′TOP mRNAs are an abundant class of mRNAs in mammalian cells that include many ribosomal protein and translation factor mRNAs. Each mRNA possesses an oligo pyrimidine sequence at or near their 5′ termini. It was proposed that LARP1 specifically promotes expression of 5′TOP mRNAs. Yeast ribosomal RNAs do not possess 5′TOP sequences, so our findings here that the yeast LARP Slf1p promotes translation of ribosomal proteins implies that there may be more than one mechanism for LARPs to promote ribosomal protein synthesis, and suggests that both human and yeast LARPs function in similar ways. It is also interesting that these proteins share some functional parallels with the eubacterial ribosomal protein S1. Similar to the LARPs, S1 is a large protein (∼68 KDa) that interacts with the small ribosomal subunit of ribosomes. S1 also binds single stranded mRNA including to a subset of mRNA 5′ leaders and can promote mRNA-ribosome interactions that activate translation initiation [34].

Analysis of the Slf1p target mRNAs has identified an AU repeat motif in the 3′UTR of Slf1 target mRNAs. We used MEME to search for motifs within the 3′ and 5′UTR regions of the mRNAs that were significantly enriched in the unstressed Slf1p RIP-Seq experiment (FDR<0.05). The UTR regions of mRNAs that were significantly decreased in the RIP-seq experiment were used as a negative control set and were not found to contain any enriched motifs. Nothing was identified for the 5′UTR region, but a 21-nt motif (mostly, alternate As and Us) was identified in the 3′UTR (Fig. 3D). This motif is not found in all sequences, but in 112 out of 414. This motif is very similar to a previously identified Pub1p motif [35] which is also an alternating AU element in the 3′UTRs of mRNAs bound by Pub1p. There was no significant overlap between our Slf1p target mRNAs to those of Pub1 [35]. A larger set of Pub1 targets have been described [36] and when comparing these to our Slf1 mRNA targets there is a significant overlap of 178 mRNAs (p = 10^−4^). However, within this overlap there is an under enrichment for the motif suggesting that the presence of the motif in the Slf1p target mRNAs is not due to shared target mRNAs with Pub1p. The motif persists in Slf1 mRNA targets after hydrogen peroxide treatment (Fig. 3D). The number of Slf1 target mRNAs containing the motif increases after oxidative stress and therefore the presence of the motif in this dataset is not simply due to Slf1p continuing to bind those target mRNAs that Slf1 binds under unstressed conditions. The physiological importance of this motif is unknown at present and will form the basis of future studies.

Taken together, these data indicate that Slf1p plays a role in mRNA-specific regulation of translation during oxidative stress conditions and is necessary to promote the translation of stress-responsive mRNAs. It does this via mRNA interactions with the well-characterised LaM [12], [24] and a novel 40S ribosome interaction region defined here. Given that Slf1p is only one of the 600 yeast proteins that are predicted to bind RNA [5] it is likely that many other RBPs will add to the complexity of mRNA-specific translational control.

## Materials and Methods

### Yeast strains and growth conditions

BY4741 was the parental strain for all deletion mutants. A BY4741 *HIS3*+ strain was generated for use as the parental strain for TAP tag immunoprecipitations. This was generated by replacing the *his3Δ1* allele in BY4741 with *HIS3*. Slf1p-TAP and Sro9p-TAP tagged strains were obtained from Open Biosystems. The BY4741 *slf1Δ* mutant was generated by replacing *SLF1* with a KanMX cassette using standard yeast genetic techniques. BY4741 *sro9Δ* was obtained from Euroscarf. All strains were grown in SCD media at 30°C to exponential phase (OD_600_ 0.5–0.7). Cultures were exposed to 0.4 mM hydrogen peroxide for 15 or 60 minutes to induce oxidative stress.

### Cloning and mutagenesis of *SLF1*

Wild-type *SLF1* and *SLF1* with point mutations within the La motif, both containing a C-terminal TAP tag, were synthesised (Epoch Life Sciences). *SLF1* constructs contained 289 nt upstream of the ATG and 234 nt downstream of the stop codon and were cloned into plasmid pRS416. pRS416-*SLF1* was used as a template to generate truncation mutations [37].

### Ribosome co-sedimentation analysis

Polyribosomal profiling was performed as previously described [38]. Briefly, *S. cerevisiae* was grown to OD_600_∼0.7, cycloheximide was added to a final concentration of 0.1 mg/ml and yeast cells were harvested by centrifugation. When cells were stressed with hydrogen peroxide, cultures were split into two 50 ml cultures and one of these was treated with hydrogen peroxide and incubated at 30°C for 15 minutes. *S. cerevisiae* were lysed in polyribosomal buffer containing cycloheximide and 2.5 OD_260_ units were loaded onto a sucrose gradient. 15–50% sucrose gradients were poured as previously described [38]. 5–25% sucrose gradients were poured in six separate fractions increasing in 5% sucrose intervals from 5–25% sucrose. For RNAse treatment, 12 units of RNAse I was added to polysome extracts and incubated for 1 h at 21°C prior to loading onto a sucrose gradient. Sucrose cushion gradients were performed as previously described [39]. For puromycin treatment, extracts were prepared in the absence of cycloheximide, and incubated with 1 mg/ml Puromycin for 10 minutes on ice prior to loading onto gradients

### Immunoprecipitation of TAP tagged proteins

TAP tagged strains were grown to exponential phase, centrifuged and washed in 3% glucose and 2x amino acids and snap frozen in liquid nitrogen. Yeast were lysed in L Buffer (20 mM Tris-HCl pH 8, 140 mM NaCl, 1 mM MgCl_2_, 0.5% NP40, 0.5 mM DTT, 1 mM PMSF, EDTA free Protease Inhibitor cocktail tablet (Roche), NaV_3_O_4_, NaF and 40 units/ml RNAsin) using a 6870 Freezer mill (Spex). Lysates were cleared by centrifuging twice at 15,000 g. Beads were prepared as previously described [40]. Beads were pre-washed three-times with L Buffer and then added to 4 mg/ml of grindate. Immunoprecipitations were performed for 20 minutes at 4°C and washed five times with L buffer containing 10 units/ml RNAsin, changing tubes at least twice during the washes and the final two washes were performed for 15 minutes each. Where RNAse treatment was performed, RNAsin was omitted from the L buffer and 200 units of RNAse I was added during the 20 minute immunoprecipitation. For RNA isolation after the final wash, the beads were resuspended in 250 µl L Buffer and treated with Trizol. The aqueous phase was mixed with 70% ethanol and the RNA was purified using the RNeasy minikit (Qiagen). For protein isolation after the final wash, protein was eluted from beads using 0.5 M sodium hydroxide. Eluted protein was concentrated using Amicon concentrator columns and analysed by immunoblotting.

### Generation of sequencing libraries

Once isolated, all RNA samples were processed in an identical manner. rRNA was depleted from the RNA samples using the Ribominus Eukaryote Kit for RNA-Seq (Invitrogen). Total RNA samples were normalised to the amount of RNA isolated from the corresponding IP sample. Depleted samples were precipitated with 2.5x volumes ethanol, 1/10^th^ volume 3 M sodium acetate and 1 µl glycogen, washed twice with 70% ethanol and re-suspended in 10 µl DEPC water. rRNA depletion was checked on a 2100 Bioanalyzer (Agilent Technologies, Palo Alto, CA) using an RNA nano-chip and the remaining RNA stored at -80°C. Sequencing libraries were generated using the whole Transcriptome Library preparation protocol provided with the SOLiD Total RNA-Seq Kit. Briefly, rRNA depleted samples were fragmented using RNase III, and subsequently cleaned up using the RiboMinus Concentration Modules (Invitrogen). Fragmentation was assessed on a 2100 Bioanalyzer using the RNA pico-chip. Fragmented RNAs were reverse transcribed and size selected on a 6% TBE-Urea gel (Novex), selecting for 150–250 nt cDNA. cDNA was then amplified and barcoded with the SOLiD RNA barcoding Kit. Samples were subsequently purified using the PureLin PCR Micro Kit (Invitrogen) and assessed on a 2100 Bioanalyzer using the High Sensitivity DNA chip. Samples were sequenced on an ABI SOLiD 4 at either The University of Manchester or at BGI.

Reads were mapped to the *S. cerevisiae* genome (genome assembly EF4 downloaded from ENSEMBL) using Bowtie; sequences were then assigned to genomic features using HTseqcount (mapping against the corresponding EF4 GTF file). Statistical significant enrichments of transcripts in the protein IPs relative to TAP-tag whole extracts were determined using the Generalized Linear Model (GLM) functionality within edgeR to produce a comparison with a paired statistical design [41] and generate gene lists at a FDR<0.05. In addition, the GLM functionality was used to measure protein specific variance between experiments through the use of an interaction model [42]. Fold changes are presented as log2 ratios of the reads per million counts (transcripts with fewer than twenty reads in each of the pertinent total extract samples were excluded from the plots). Sequencing data are publicly available on ArrayExpress, E-MTAB-2567 (Slf1p) and E-MTAB-2568 (Sro9p). Functional categorisation of mRNAs and proteins was performed using MIPS Functional Catalogue (mips.helmholtz-muenchen.de/proj/funcatDB/).

5′ and 3′ UTR regions of mRNAs bound by Slf1p in the presence or absence of hydrogen peroxide were searched for common sequence motifs using the MEME Suite [43]. In all cases, the equivalent regions of the depleted mRNAs in the RIP-seq experiment were used as a negative set. In an additional control, the letters in the positive set were shuffled in order to check that the motif did not come out because of the relative frequencies of nucleotides.

### Transcriptomic analysis

The parental and mutant strains were grown in SCD to exponential phase and treated as described above. RNA was isolated from cleared lysates using Trizol and used to generate sequencing libraries. To enable a comparison of the transcriptomes of both mutants, transcriptomes were binned into 0.25-fold bins based on fold enrichment above the parental strain. These data were then expressed as a percentage frequency of transcripts within each bin. Data were not filtered on FDR prior to binning.

### Label-free protein quantification

Quintuplicate repeats of the wild-type and *slf1Δ* strains were grown in SCD media to exponential phase, split in two, and half treated with 0.4 mM hydrogen peroxide for 1 h. Cultures were harvested, washed in 3% glucose with 2x amino acids and snap frozen in liquid nitrogen. Cell pellets were lysed using the 6870 freezer mill (Spex) into 8 ml of 25 mM ammonium bicarbonate buffer containing a protease inhibitor cocktail tablet (Roche). Ground samples were defrosted, cleared by centrifugation (15,000 *g* 10 minutes), and 100 µg of cleared lysate was diluted to a final volume of 160 µl containing 1% (w/v) RapiGest (Waters Corporation). Samples were incubated at 80°C for 10 minutes, reduced using a final concentration of 3.5 mM DTT in 25 mM ammonium bicarbonate and incubated at 60°C for 10 minutes. Iodoacetamide was added to a final concentration of 10 mM and incubated at room temperature for 30 minutes. A final concentration of 0.01 µg/µl trypsin in 10 mM acetic acid was added and samples were digested for 4.5 h at 37°C. Hydrochloric acid was added to a final concentration of 13 mM and a second identical trypsin digest was performed overnight at 37°C. 0.5 µl of trifluoroacetic acid was added and incubated at 37°C for 2 h. 7.5 µl of acetonitrile:water (2∶1) was added and incubated at 4°C for 2 h and centrifuged at 13,000 *g* for 15 minutes. Supernatant was removed and desalted using OLIG R3 reversed-phase media on a microplate system. Peptides were eluted in three cycles of 50% acetonitrile and dried by vacuum centrifugation, and reconstituted to 10 µL with 5% acetonitrile and 0.1% formic acid.

Digested samples were analysed by LC-MS/MS using an UltiMate 3000 Rapid Separation LC (RSLC, Dionex Corporation, Sunnyvale, CA) coupled to an Orbitrap Elite (Thermo Fisher Scientific, Waltham, MA) mass spectrometer. Peptide mixtures were separated using a gradient from 92% A (0.1% FA in water) and 8% B (0.1% FA in acetonitrile) to 33% B, in 44 min at 300 nL min^−1^, using a 250 mm×75 µm i.d. 1.7 mM BEH C18, analytical column (Waters). Peptides were selected for fragmentation automatically by data dependant analysis.

The acquired MS data from the five replicates were analysed using Progenesis LC-MS (v4.1, Nonlinear Dynamics). The retention times in each sample were aligned using one LC-MS run as a reference, then the “Automatic Alignment” algorithim was used to create maximal overlay of the two-dimensional feature maps. Features with charges ≥+5 were masked and excluded from further analyses, as were features with less than 3 isotope peaks. The resulting peaklists were searched against the *Saccharomyces* Genome Database (SGD, version 3^rd^ February 2011) proteome using Mascot v2.4 (Matrix Science). Search parameters included a precursor tolerance of 5 ppm and a fragment tolerance of 0.5 Da. Enzyme specificity was set to trypsin and one missed cleavage was allowed. Carbamidomethyl modification of cysteine was set as a fixed modification while methionine oxidation was set to variable. The Mascot results were imported into Progenesis LC-MS for annotation of peptide peaks. The mass spectrometry proteomics data have been deposited to the ProteomeXchange Consortium (http://www.proteomexchange.org) via the PRIDE partner repository with the dataset identifier PXD000887 and DOI 10.6019/PXD000887.

## Supporting Information

S1 Fig *slf1Δ* and *sro9Δ* transcriptome analysis. (**A**) Volcano plots showing transcripts that increase or decrease in abundance in an *slf1Δ* (blue) and an *sro9Δ* (red) strain. (**B**) Comparison of transcripts that change in an *slf1Δ* strain (x-axis) and an *sro9Δ* strain (y-axis). The scatterplot is coloured to correspond with those scatterplots in (A). Crossover between datasets is tabulated and coloured (green and brown spots) to correspond with the scatterplot.
(TIF)

S2 Fig Slf1p and Sro9p RIP Seq analysis. (**A**) Venn diagrams are shown comparing our Slf1p and Sro9p RIP-Seq data with microarray data from Schenk *et al* (2012). (**B**) Scatterplots are coloured to correspond with the colours in the Venn diagrams above. Why both approaches identify distinct but overlapping members of the same functional groups is not clear. However, it is not the case that those transcripts that are enriched in both our study and the Schenk *et al* study are simply the greatest confidence targets, these plots show that the intersect between the Seq and array experiments are not due to those mRNA targets with the highest P-values/FDR.
(TIF)

S3 Fig Comparison of RIP-Seq data with transcriptome data. Venn diagrams are shown comparing the Slf1p (**A**) and Sro9p (**B**) RIP Seq data with the *slf1Δ* and *sro9Δ* transcriptome data.
(TIF)

S4 Fig Slf1p targets decrease in steady state levels in an *slf1Δ* strain. Transcript abundance, determined by SOLiD sequencing, of *slf1Δ* (**A**) and *sro9Δ* (**B**) mutant strains was compared to the parental strain and expressed as Log2 fold enrichment. Transcriptomes were split into bins (0.25 fold/bin) and expressed as a percentage of transcripts in each bin. The same analysis was also applied to the Slf1p and Sro9p targets identified by RIP-Seq. An increasing FDR cut-off was applied to the RIP Seq identified targets selecting for higher confidence targets. In an *slf1Δ* strain the abundance of targets decreases as confidence increases (A). This does not happen in an *sro9Δ* strain (B). RIP Seq targets are red and the genome is blue.
(TIF)

S5 Fig Stress sensitivity of *slf1Δ* and *sro9Δ* mutant strains. (**A**) The indicated strains were tested for stress sensitivity by growth at low (16°C) and high (37°C) temperatures, low pH (5) and YEPD plates containing 1 M NaCl, 18 mM copper (Cu) and 4 mM hydrogen peroxide. (**B**) A plasmid-borne copy of *SLF1* complements the hydrogen peroxide sensitivity of a *slf1Δ* mutant strain. (**C**) Polyribosome traces are shown for the wild-type and *sro9Δ* strain treated with hydrogen peroxide.
(TIF)

S6 Fig Translation initiation is less inhibited in an *slf1Δ* strain in response to hydrogen peroxide stress. Polyribosomal profiles are shown for the *slf1Δ* strain and the parental strain after hydrogen peroxide treatments for 15 minutes. The hydrogen peroxide concentration is indicated above each polyribosomal trace (mM) and the monosome:polysome ratio (M:P) is shown. These M:P data were used to generate Fig. 3B
(TIF)

S7 Fig Comparison of translation efficiency (TE) with Slf1p-RIP-Seq and proteomic data. A recent genome-wide ribosome profiling study has provided translation efficiency (TE) data (amount of footprint normalized to underlying mRNA abundance) following treatments with 0.2 mM hydrogen peroxide for five or 30 minutes [32]. We compared this dataset with our Slf1p-RIP-Seq and proteomics analyses which treated yeast cells with 0.4 mM hydrogen peroxide for 15 minutes. Only transcripts or proteins with an associated FDR<0.05 were considered to be significantly enriched (up) or depleted (down); the rest of the transcripts or proteins were classified as not changing. Distributions are shown as box and whisker plots, with a 95% confidence interval around the median represented by a notch. Thus, if two notches do not overlap, we can roughly say that the two medians are different. The differentially regulated transcripts and proteins show different distributions of translational efficiency (Kruskal-Wallis test; FDR<0.01), apart from for the short response TE (5 minutes), compared with the proteomics experiment.
(TIF)

S1 Table Transcriptome changes in the *slf1Δ* mutant strain compared with a wild-type strain.
(XLSX)

S2 Table Transcriptome changes in the *sro9Δ* mutant strain compared with a wild-type strain.
(XLSX)

S3 Table Identication of Slf1p mRNA targets by RIP-Seq analysis.
(XLSX)

S4 Table Identication of Sro9p mRNA targets by RIP-Seq analysis.
(XLSX)

S5 Table Identication of Slf1p mRNA targets following treatment with hydrogen peroxide by RIP-Seq analysis.
(XLSX)

S6 Table Label Free Poteomics in the wild-type strain before (WT) or after 60 min Hydrogen peroxide treatment (WT H_2_O_2_).
(XLSX)

S7 Table Label Free Poteomics in the *slf1Δ* strain before (*slf1*) or after 60 min Hydrogen peroxide treatment (*slf1* H_2_O_2_).
(XLSX)
