# Supplementary figures and images for: The Yeast La Related Protein Slf1p Is a Key Activator of Translation during the Oxidative Stress Response
Source: PLoS Genet. 2015 Jan 8;11(1):e1004903. doi: 10.1371/journal.pgen.1004903 (PMC4287443; doi:10.1371/journal.pgen.1004903)

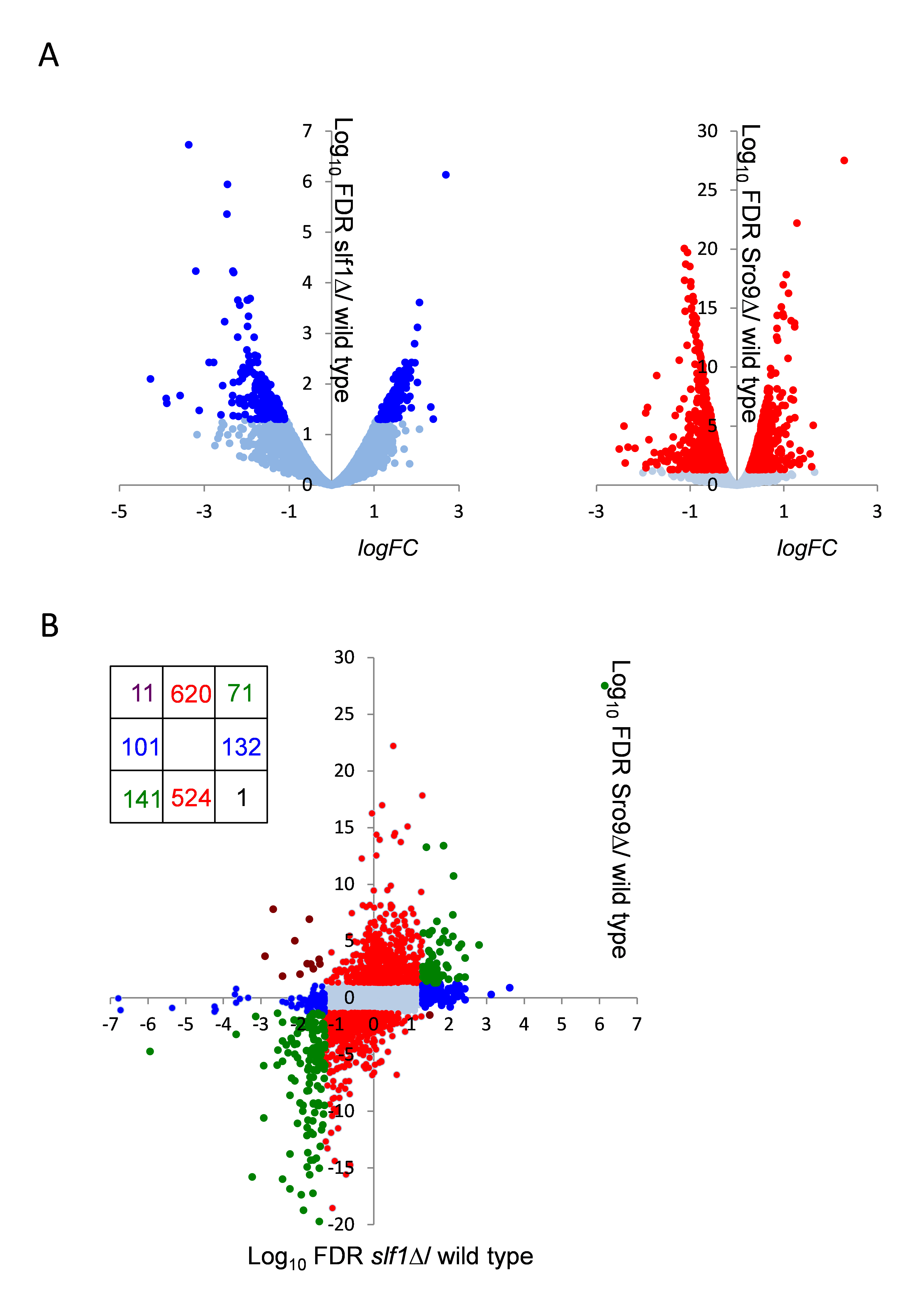

Supplement: S1 Fig — slf1Δ and sro9Δ transcriptome analysis. (A) Volcano plots showing transcripts that increase or decrease in abundance in an slf1Δ (blue) and an sro9Δ (red) strain. (B) Comparison of transcripts that change in an slf1Δ strain (x-axis) and an sro9Δ strain (y-axis). The scatterplot is coloured to correspond with those scatterplots in (A). Crossover between datasets is tabulated and coloured (green and brown spots) to correspond with the scatterplot. (TIF) [file pgen.1004903.s001.tif]

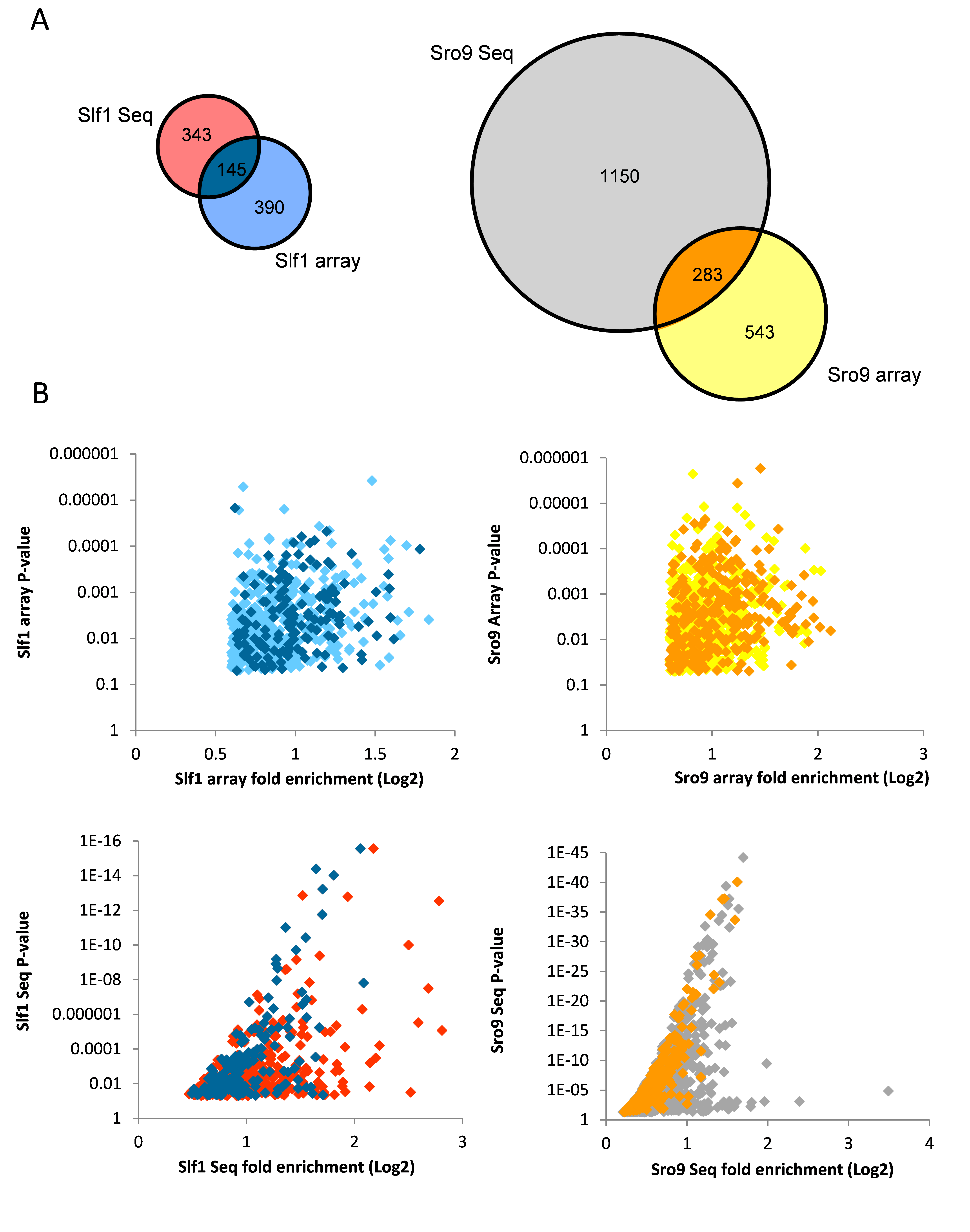

Supplement: S2 Fig — Slf1p and Sro9p RIP Seq analysis. (A) Venn diagrams are shown comparing our Slf1p and Sro9p RIP-Seq data with microarray data from Schenk et al (2012). (B) Scatterplots are coloured to correspond with the colours in the Venn diagrams above. Why both approaches identify distinct but overlapping members of the same functional groups is not clear. However, it is not the case that those transcripts that are enriched in both our study and the Schenk et al study are simply the greatest confidence targets, these plots show that the intersect between the Seq and array experiments are not due to those mRNA targets with the highest P-values/FDR. (TIF) [file pgen.1004903.s002.tif]

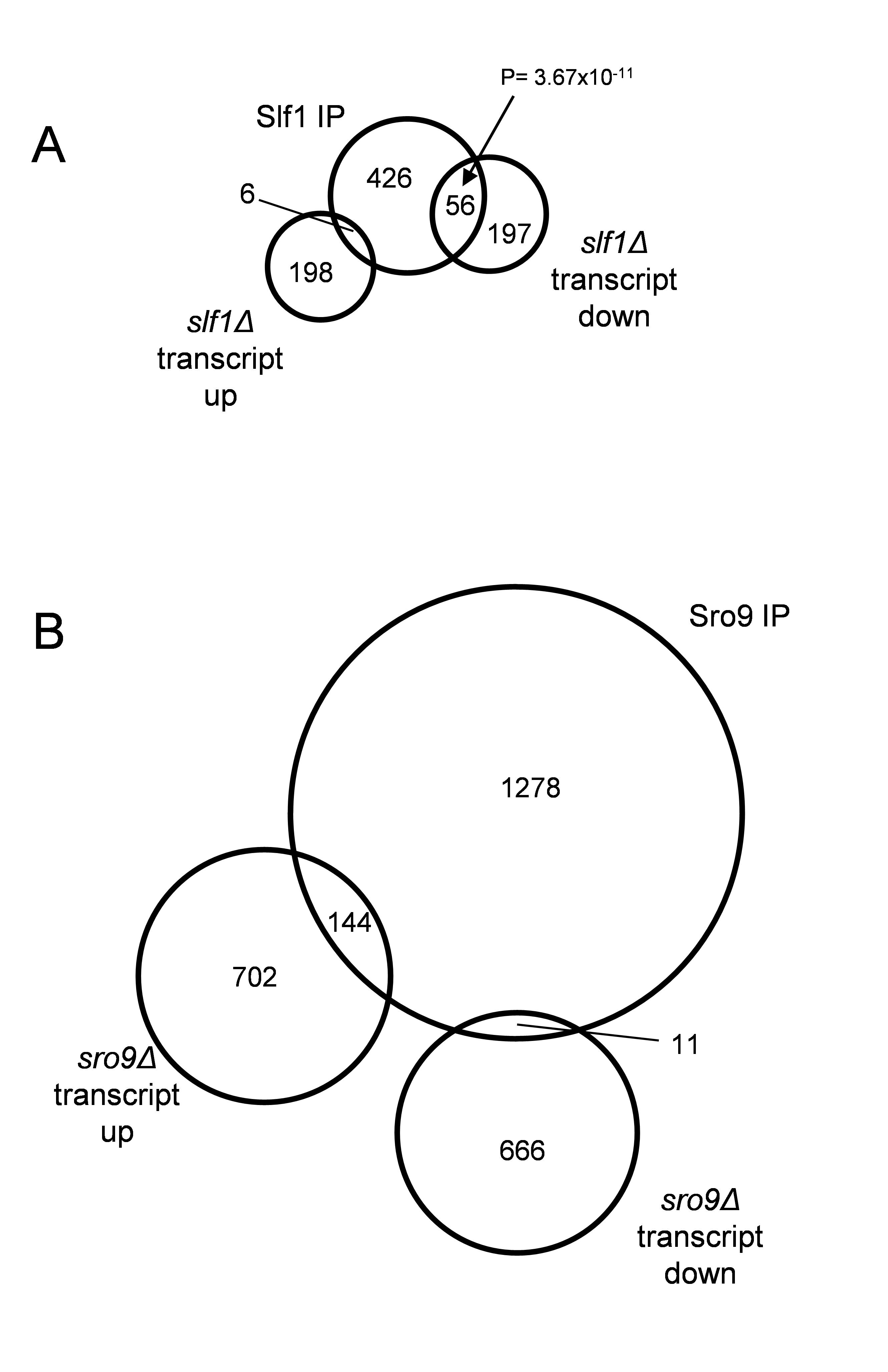

Supplement: S3 Fig — Comparison of RIP-Seq data with transcriptome data. Venn diagrams are shown comparing the Slf1p (A) and Sro9p (B) RIP Seq data with the slf1Δ and sro9Δ transcriptome data. (TIF) [file pgen.1004903.s003.tif]

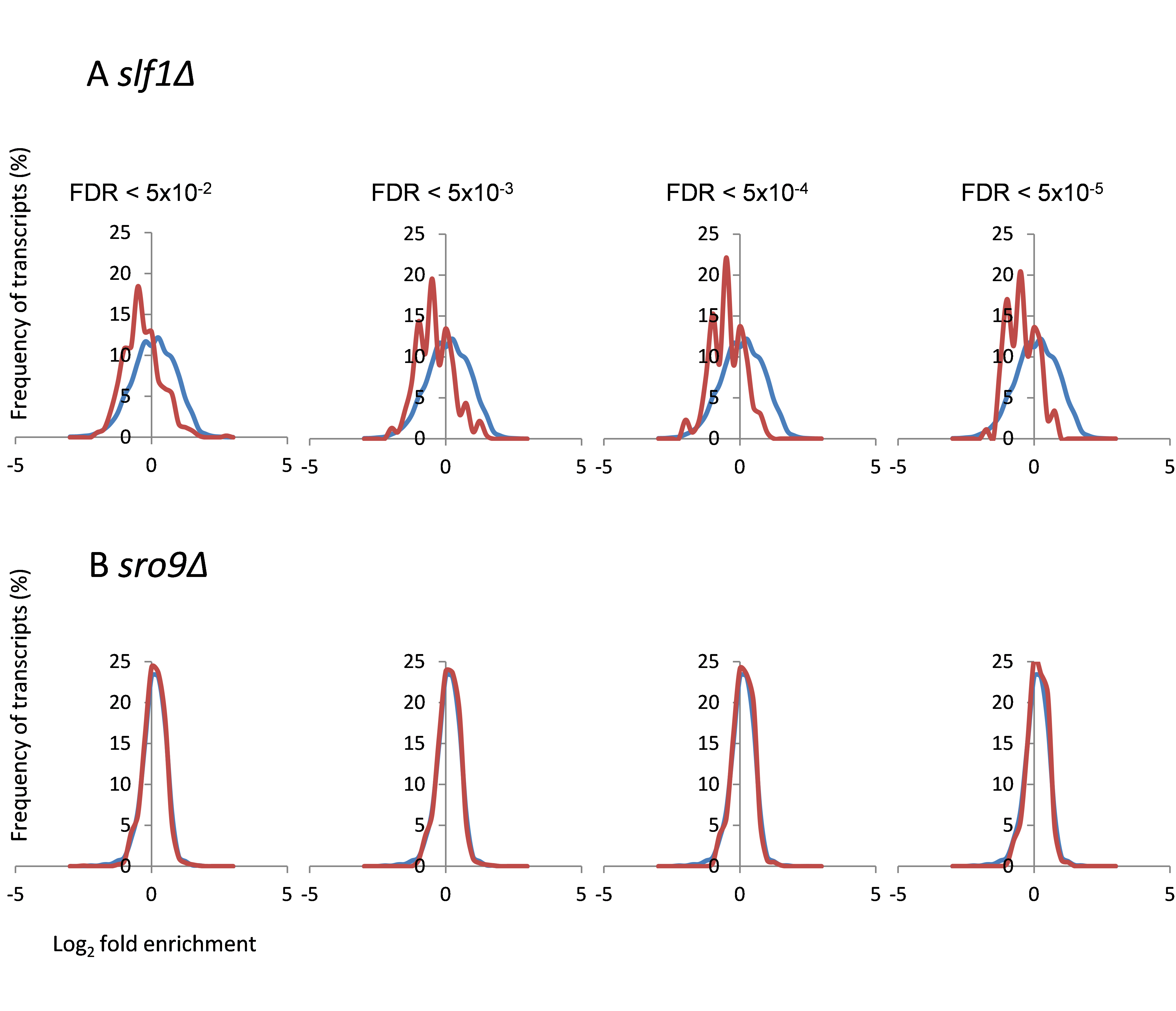

Supplement: S4 Fig — Slf1p targets decrease in steady state levels in an slf1Δ strain. Transcript abundance, determined by SOLiD sequencing, of slf1Δ (A) and sro9Δ (B) mutant strains was compared to the parental strain and expressed as Log2 fold enrichment. Transcriptomes were split into bins (0.25 fold/bin) and expressed as a percentage of transcripts in each bin. The same analysis was also applied to the Slf1p and Sro9p targets identified by RIP-Seq. An increasing FDR cut-off was applied to the RIP Seq identified targets selecting for higher confidence targets. In an slf1Δ strain the abundance of targets decreases as confidence increases (A). This does not happen in an sro9Δ strain (B). RIP Seq targets are red and the genome is blue. (TIF) [file pgen.1004903.s004.tif]

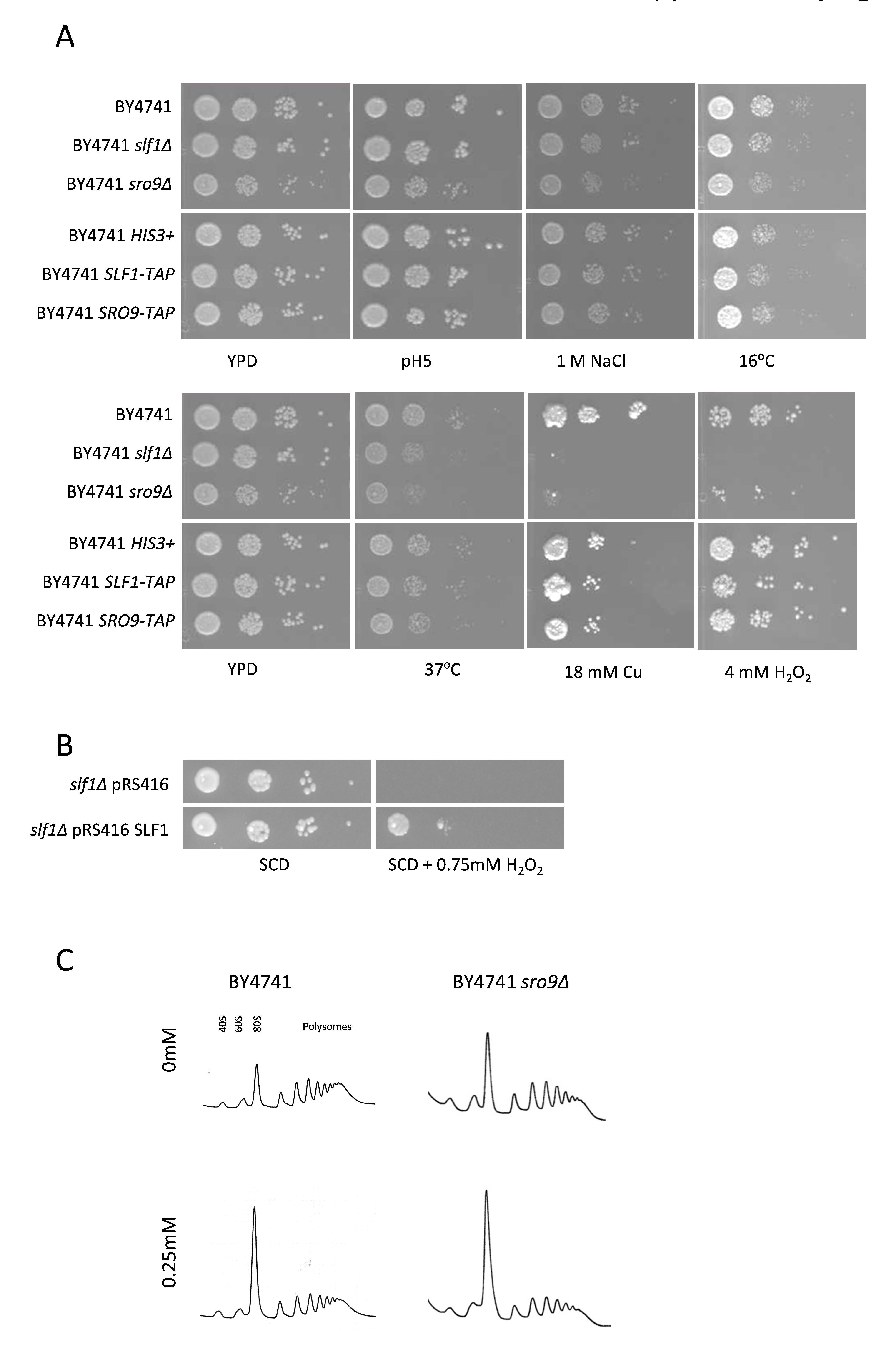

Supplement: S5 Fig — Stress sensitivity of slf1Δ and sro9Δ mutant strains. (A) The indicated strains were tested for stress sensitivity by growth at low (16°C) and high (37°C) temperatures, low pH (5) and YEPD plates containing 1 M NaCl, 18 mM copper (Cu) and 4 mM hydrogen peroxide. (B) A plasmid-borne copy of SLF1 complements the hydrogen peroxide sensitivity of a slf1Δ mutant strain. (C) Polyribosome traces are shown for the wild-type and sro9Δ strain treated with hydrogen peroxide. (TIF) [file pgen.1004903.s005.tif]

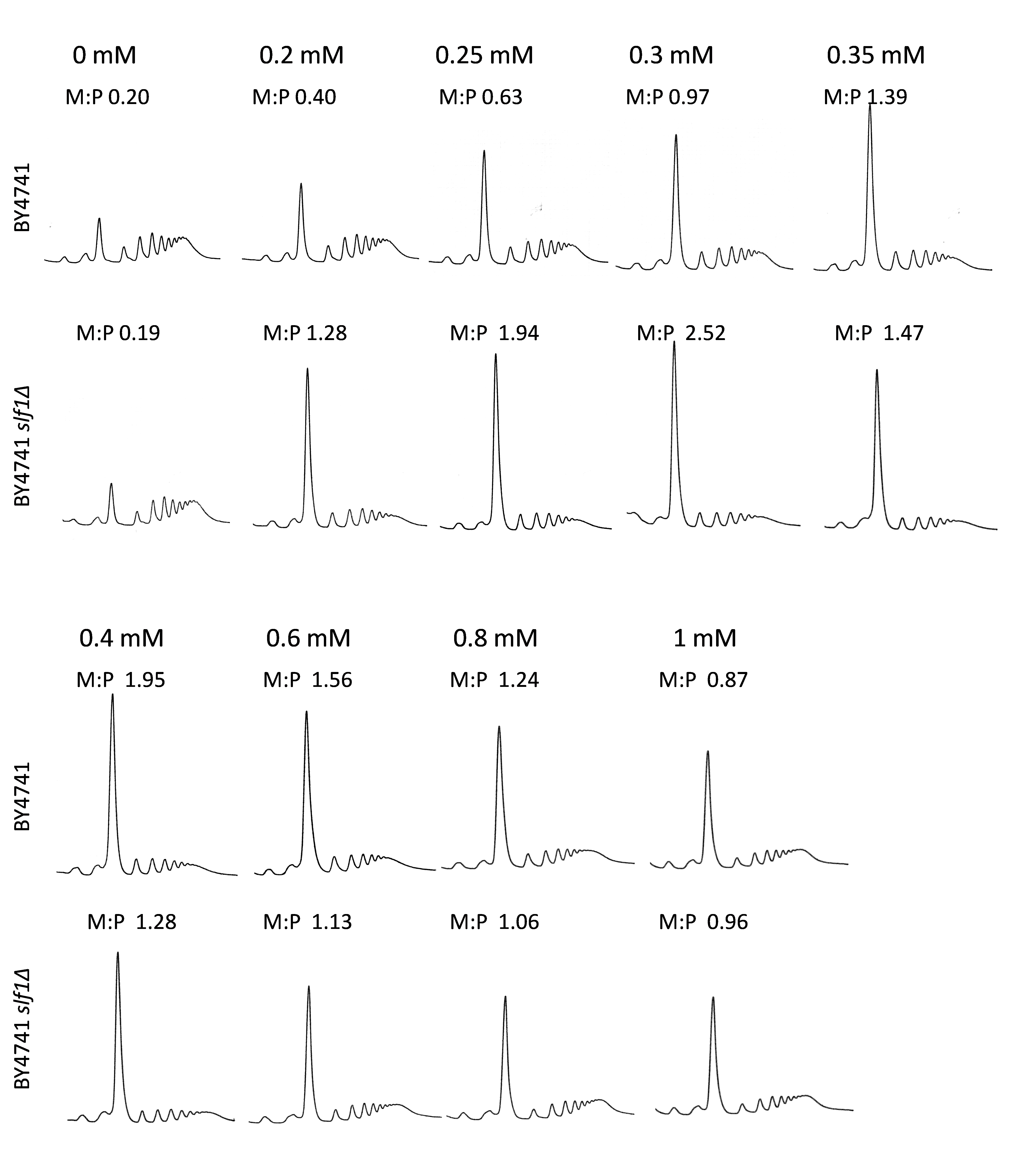

Supplement: S6 Fig — Translation initiation is less inhibited in an slf1Δ strain in response to hydrogen peroxide stress. Polyribosomal profiles are shown for the slf1Δ strain and the parental strain after hydrogen peroxide treatments for 15 minutes. The hydrogen peroxide concentration is indicated above each polyribosomal trace (mM) and the monosome:polysome ratio (M:P) is shown. These M:P data were used to generate Fig. 3B (TIF) [file pgen.1004903.s006.tif]

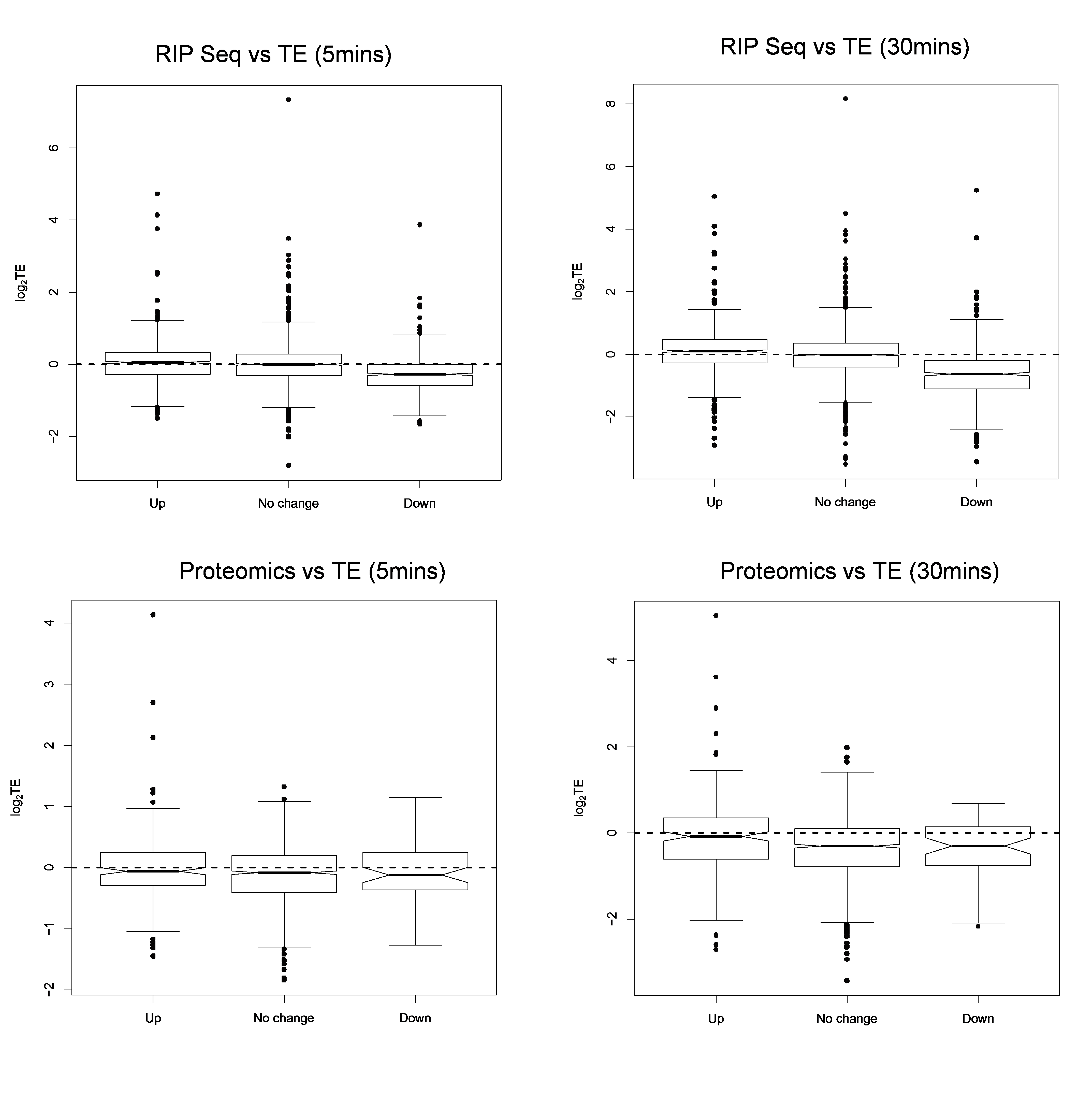

Supplement: S7 Fig — Comparison of translation efficiency (TE) with Slf1p-RIP-Seq and proteomic data. A recent genome-wide ribosome profiling study has provided translation efficiency (TE) data (amount of footprint normalized to underlying mRNA abundance) following treatments with 0.2 mM hydrogen peroxide for five or 30 minutes [32]. We compared this dataset with our Slf1p-RIP-Seq and proteomics analyses which treated yeast cells with 0.4 mM hydrogen peroxide for 15 minutes. Only transcripts or proteins with an associated FDR<0.05 were considered to be significantly enriched (up) or depleted (down); the rest of the transcripts or proteins were classified as not changing. Distributions are shown as box and whisker plots, with a 95% confidence interval around the median represented by a notch. Thus, if two notches do not overlap, we can roughly say that the two medians are different. The differentially regulated transcripts and proteins show different distributions of translational efficiency (Kruskal-Wallis test; FDR<0.01), apart from for the short response TE (5 minutes), compared with the proteomics experiment. (TIF) [file pgen.1004903.s007.tif]
